# A Large-Area Uniform Three-Dimensional Covalent Organic Framework Membrane for Stabilizing Li-Metal Electrodes via Solvation Cages

**DOI:** 10.34133/research.0926

**Published:** 2025-10-09

**Authors:** Zhuozhuo Tang, Jia Chen, Da Zhu, Li Sheng, Yang Yang, Kai Yang, Jianlong Wang, Yaping Tang, Xiangming He, Hong Xu

**Affiliations:** Institute of Nuclear and New Energy Technology, Tsinghua University, Beijing 100084, P. R. China.

## Abstract

Covalent organic frameworks (COFs), known for their ordered structures, hold promise as ion-conducting materials in cells/batteries. Nevertheless, the rigid cross-linking of porous materials prevents them from being processed into membranes, while composite membranes weaken the material’s conductivity advantage due to phase interruptions. Here, we report a phase-continuous 3-dimensional COF (3D-COF) membrane with a large size of 15 cm × 25 cm, fabricated via in situ interfacial engineering. The COF membranes possessed a non-interpenetrating dia topology that facilitated 3D continuous ionic pathways at the molecular level. Further, the hydroxyl and imine groups on the framework could form Li^+^-solvation cages, providing the hydrogen-bonding locking sites that facilitate the conversion of the Li-solvates into more readily reducible species. Combined with the dense nanoporous feature, this 3D-COF membrane was found to be very effective in inhibiting Li-dendrites and parasitic reactions and demonstrated a stabilizing effect and good cycling performance in the Li|NMC622 batteries.

## Introduction

Covalent organic frameworks (COFs) are crystalline porous materials in which organic building blocks are integrated with atomic precision to create predesigned skeletons and nanopores [1–3]. The porous feature endows COFs with great potential for hydrogen/hydrocarbon storage [4–7], CO_2_ capture [8–13], water harvesting [14–17], liquid-phase adsorption [18–20], and catalysis [21–24]. Besides, the well-ordered channel structures, which originated from their unique crystalline features, have caused tremendous interest in proton/hydroxide conduction and Li^+^-ion transport [25–29]. This is because the sophisticated and feature-tunable nanochannel structures found in COFs have long been sought after and are nearly unattainable in polymer-based ion-conducting materials. From the perspective of COF materials, these ion transport-related utilizations are also the direct use of the crystalline and pore designable feature of COFs, which distinguishes COF materials from other amorphous porous materials. However, from the perspective of fuel cells or lithium-ion batteries, membrane fabrication is a prerequisite that needs to be addressed before COF-like materials can be used as ion-conducting materials in cells/batteries.

COFs are a highly cross-linked network, which is the origin of the porous nature of the material, while the ordering of such a network is the source of its crystallinity [30]. However, the highly cross-linked network also means that the material cannot be dissolved in solvents or melted at elevated temperatures, precluding the possibility of solution casting and melt pouring to produce membranes, which are the most commonly used means of film/membrane fabrication [31,32]. Faced with the challenge of fabricating membranes from COFs [and also metal-organic frameworks (MOFs) [33–36]] and seeking the benefits of nanoporosity, polymer bonding is often used in the field, but this leads to the disconnection of the ion channels in the COF/MOF material, largely diminishing the advantages of the material’s sophisticated nanochannels [37–39]. Nevertheless, revisiting the next-to-impossible challenge of constructing crystals from polymers at the time of COF’s inception, the use of in situ reversible polymerization reactions became the game changer in controlling covalent linkages in 2-dimensional (2D) and 3D covalent organic solids [40,41]. Through the rational design of in situ polymerization approaches and organic building blocks, the membrane fabrication challenge of COF is not insurmountable.

Here, we report a substrate-assisted interfacial polymerization approach to easily prepare a uniform, large-area, and microcontinuous 3D-COF membrane, and demonstrate its great application in Li-metal battery to stabilizing Li-metal anode through the electron-rich sites integrated on its channels. During the interface polymerization, porous polymer behaves as a COF growth substrate, regionally enriching the COF building blocks and promoting their condensation reactions. Meanwhile, the hydroxyl and imine groups, which provide hydrogen bond locking during COF synthesis, facilitate the formation of a continuous, large-area COF membrane (15 cm × 25 cm). The COF membrane has sufficient strength and considerable thickness (~4 μm) to be peeled from the substrate to obtain a self-standing membrane. Unlike traditional 3D-COFs prepared by the solvothermal method, this 3D-COF membrane features a non-interpenetrating dia topology, which promotes the formation of 3D continuous ionic pathways at the molecular level, enabling rapid ion transport—a crucial characteristic for lithium-ion batteries. Furthermore, as a separator in the Li-metal battery, the 3D-COF membrane shows an obvious Li-metal anode stabilization effect, which suppresses parasitic reactions and Li-dendrite growth. This was because of the following reasons. Firstly, the hydroxyl and imine groups on the framework can act as Li^+^-solvation cages, facilitating the conversion of the Li-solvates to more readily reducible species [e.g., Li(EC)_4_^+^ to Li(EC)_3_^+^]. Secondly, this 3D-COF membrane features dense nanoporous, which can realize the uniform deposition of lithium on the electrode surface. Therefore, incorporating the dense nanoporous membrane and the Li^+^-solvation cages, the 3D-COF membrane paves an effective method to improve the cycling performance of lithium metal batteries.

## Results

### Preparation of 3D-COF membrane

**Fig. 1.**
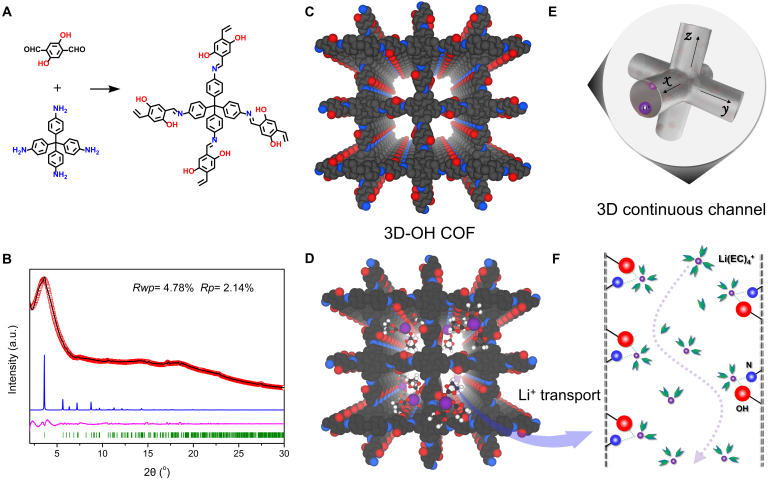
Synthesis and structure of 3D-COF. (A) Synthesis of 3D-COF. (B) XRD pattern of the 3D-COF membrane (XRD refinement, experimental pattern: red curve, Pawley refined pattern: black curve, calculated pattern from the nonfold dia model: blue curve, difference pattern: purple curve, Bragg positions: green). (C) Structure of 3D-COF (carbon, black; oxygen, red; nitrogen, blue). (D) Transport of Li^+^-solvate in 3D-COF (carbon, black; oxygen, red; nitrogen, blue; Li, purple). (E) Schematic diagram of 3D channels in 3D-COF membrane. (F) Desolvation processes of Li^+^ within 3D-COF channels (red ball: hydroxyl; blue ball: imine group).

Through oil/water 2-phase interfacial polymerization, COF nanosheets can be formed, and these nanosheets (usually 2D-COFs) can produce small-sized COF membranes by the filtration and restacking method [42], and exhibit great potential in proton conduction [43,44]. Compared to the widely studied 2D COFs, 3D structures offer better connectivity, facilitating the rapid passage of ions and solvent molecules [45]. Recently, we found that placing a porous polymer membrane on the 2-phase interface cloud regionally enriches COF monomers and promotes their condensation reaction [41]. Self-standing membranes are of scientific importance for producing membranes from rigid porous materials, but their mechanical strength and flexibility are limited, which is not conducive to further application exploration. Thus, here, we used a porous polypropylene membrane (Celgard2500, commonly used as a separator in lithium-ion batteries; abbreviated as PP) with stretched pores as the substrate for interfacial polymerization and as the basal membrane to provide flexibility for subsequent lithium-metal battery assembly. Meanwhile, considering the low-temperature conditions of the interfacial polymerization, which leads to poor reversibility of the COF formation reaction, we introduced hydroxyl groups at the ortho-positions of the aldehyde monomer to promote the high-quality growth of COF through the hydrogen bond locking mechanism. In the interfacial in situ engineering, hydroxyl-containing monomers and tetrahedral aldehydes were condensed to form 3D-COF, as illustrated in Fig. 1. Moreover, this method exhibits good generality and can be further extended to the synthesis of 3D-COF membrane incorporating other functional groups as shown in Fig. S1.

In the fabrication of the COF membrane, tetra-(4-anilyl)-methane (TAM) and 2,5-dihydroxyterephthalaldehyde (DHTA) were dispersed in deionized water and n-octanoic solution, respectively. A 6 M acetic acid solution was added to the aqueous phase, which was then placed at the bottom of a flat glass dish. The PP membrane was placed on top of the aqueous phase, and then the oil phase was added. A clear interface was formed between the oil and aqueous phase, where the PP membrane was stably positioned. After being left at room temperature (25°C) for 3 to 5 d, a uniform yellow COF membrane formed on the surface of the PP membrane, named 3D-COF@PP, as a 3D-COF separator in Li batteries. The fabrication process of the 3D-COF separator is illustrated in Fig. S2. The stress–strain tests (Fig. S3) demonstrate that the 3D-COF membrane exhibits excellent mechanical properties. The 3D-COF separator exhibits a certain degree of flexibility and mechanical strength, making it suitable for bending and processing as a separator for coin cells.

### Characterization of 3D-COF separator

Due to the nanoconfinement engineering, the growth of 3D-COFs at the interface differs from the isotropic growth in solvothermal methods [46,47]. The interface restricts COF growth in the vertical direction, resulting in a 3D-COF with a non-interpenetrated dia topology, as shown in Fig. 1C and Fig. S4. The crystal structure of 3D-COF was observed using x-ray diffraction (XRD), as depicted in Fig. 1B. The N_2_ adsorption–desorption isotherm and pore size distribution of 3D-COF are shown in Figs. S5 and S6. Compared to the traditional 7-fold structures synthesized via solvothermal methods (Figs. S7 to S10), this non-interpenetrated structure tends to have higher porosity and connectivity, facilitating the smooth passage of ions and solvent molecules (Fig. S11). Additionally, the hydroxyl groups at the ortho-positions of the aldehyde monomer promote the high-quality growth of COF through the hydrogen bond locking mechanism.

Meanwhile, the confined growth on the interface leads to a distinct microstructure of the 3D-COF membrane compared to particles. Unlike the conical COF particles obtained through solvothermal synthesis (Fig. S12), the 3D-COF formed via interfacial in situ polymerization is a smooth spherical structure (Fig. S13). These small spheres can stack uniformly and tightly together, ultimately forming continuous membranes. The morphology of the 3D-COF membrane maintains good crystallinity, with clear lattice fringes visible in the transmission electron microscopy images (Fig. S13). In the cross polarization-magic angle spinning ^13^C nuclear magnetic resonance (CP-MAS ^13^C NMR) spectrum, a strong carbon signal for C–O at 150 parts per million (ppm) and a peak at 160 ppm corresponding to C=N were observed (Fig. S14). The Fourier transform infrared (FTIR) spectrum of the 3D-COF membrane shows a characteristic peak for the C=N bond at 1,560 cm^−1^, indicating the presence of imine bonds (Fig. S15). Additionally, the thermogravimetric analysis (TGA) results reveal that the 3D-COF membrane exhibits excellent thermal stability, with a mass loss of less than 5% at 356 °C, fully meeting the requirements for battery applications (Fig. S16).

**Fig. 2.**
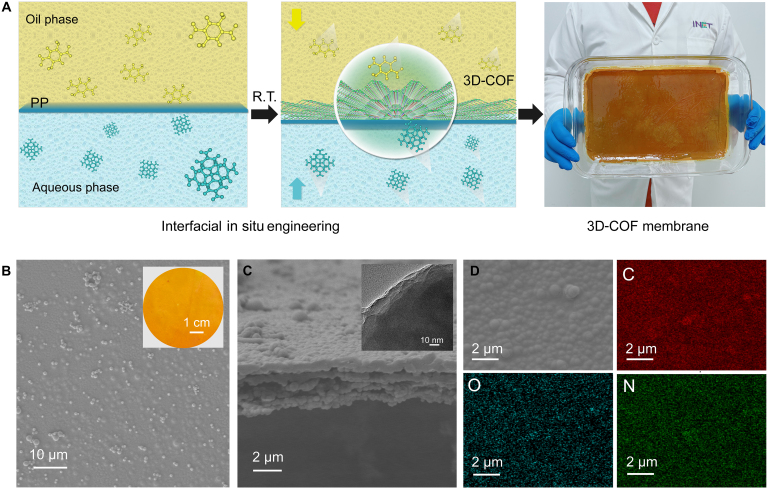
Preparation and morphology of 3D-COF membrane. (A) Schematic of the preparation of 3D-COF membrane. (B) SEM image of 3D-COF separator (3D-COF side, inset: optical picture of 3D-COF). (C) Cross-section SEM of the 3D-COF membrane. (D) EDS mapping of 3D-COF separator: C, N, O elemental distribution.

It’s worth noting that this interfacial in situ polymerization method resulted in a uniform and ordered nanoporous COF membrane. The synthesis process of the 3D-COF membrane via the in situ polymerization method is shown in Fig. 2A. The confined growth forced the COF to assemble laterally, inhibiting vertical growth at the interface (with an aspect ratio of up to 20,000:1). Compared to the COF particle-based membranes obtained through traditional coating methods, this in situ polymerization avoids membrane inhomogeneity caused by particle gaps. Unlike the original PP membrane in appearance (Fig. S17, inset photo), the 3D-COF crystalline membrane is uniformly dense (Fig. 2B, inset photo) due to its continuous microlevel structure.

Furthermore, scanning electron microscope (SEM) images show that the 3D-COF membrane is dense, uniform, and free of significant defects, with a very smooth surface, confirming it as a complete and continuous crystalline membrane with a thickness of approximately 4 μm (Fig. 2C). To evaluate the uniformity of the as-prepared 3D-COF membrane, 5 different points were evenly selected across its surface, and cross-sectional SEM images were recorded. As shown in Fig. S18, the cross-sectional thickness at each position is approximately 4 μm, exhibiting excellent uniformity. These results demonstrate that the in situ interfacial polymerization method enables the fabrication of large-area COF membranes with high uniformity. The energy-dispersive x-ray spectroscopy (EDS) mapping results reveal a clear and uniform distribution of oxygen (O) elements in the 3D-COF separator, consistent with the distribution of carbon (C) and nitrogen (N) (Fig. 2D). This uniform and consistent COF membrane on the PP membrane, achieved through in situ polymerization, is challenging to attain with a nanoporous membrane prepared using COF/MOF powders via coating methods. The porous particles inevitably cause significant thickness and noticeable uneven distribution, potentially increasing the ohmic resistance of the battery, which is detrimental to ion transport through the separator.

This interfacial in situ polymerization method for preparing 3D-COF membranes is also feasible for preparing free-standing membranes (Fig. S19). The 3D-COF membrane was then peeled off from the substrate, retaining good mechanical strength and ductility. This substrate-assisted interfacial polymerization engineering provides an alternative method for preparing free-standing COF membranes. However, due to the stringent mechanical performance requirements during the lithium battery assembly process, the 4-μm COF membrane prepared via interfacial polymerization is too brittle for use in battery assembly. Nonetheless, this self-standing membrane may hold potential for applications in proton/hydroxide ion conduction, gas separation, and other related fields.

### Electrochemical properties

**Fig. 3.**
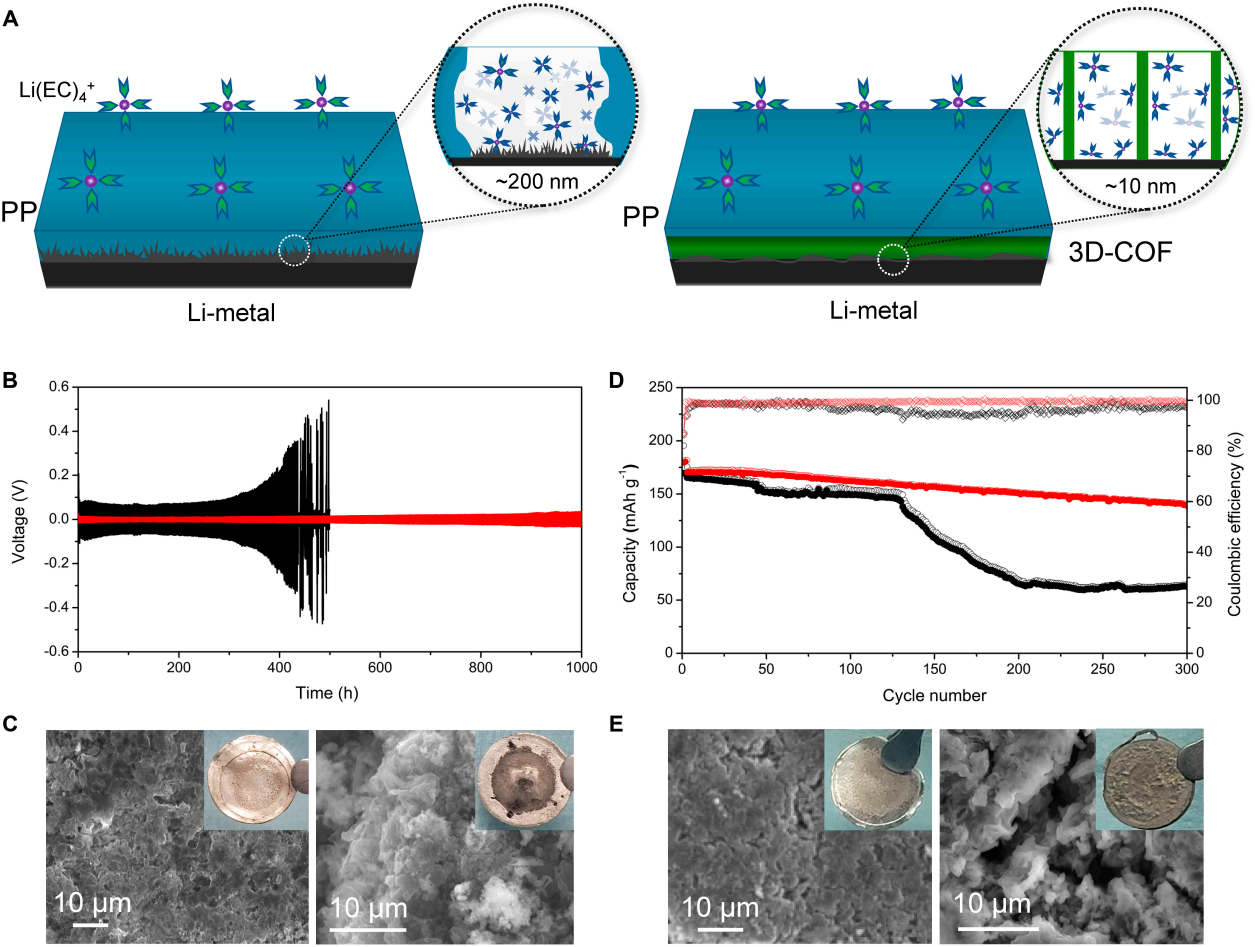
Electrochemical performance and morphology of cycled lithium metal in Li-symmetric cells and Li|NMC622 cells. (A) Schematic diagram of Li^+^ transport in 3D-COF and PP separator. (B) Initial voltage profiles of the PP separator (black)- and 3D-COF (red)-based cells at a fixed current density of 1 mA cm^−2^. (C) SEM images of the lithium metal after 500 h in Li-symmetric cells. Insets are photos of the corresponding lithium metal electrode (left: 3D-COF separator, right: PP separator). (D) Cycling stability and Coulombic efficiency of Li|NMC622 cells with the PP separator (black) and 3D-COF (red) at a 1-C rate; the voltage range of charge and discharge is 2.7 to 4.6 V. (E) SEM images of Li metal after 300 cycles in Li|NMC622 cells. Insets are photos of the corresponding lithium metal electrode (left: 3D-COF separator, right: PP separator).

We assembled the 3D-COF separator into coin cells to measure their electrochemical performance (Fig. 3). To evaluate the stability of the Li-metal electrode and the issue of Li^0^ deposition on the electrode surface, we used a commercial Li^+^ battery electrolyte [a mixture of lithium hexafluorophosphate (LiPF_6_) dissolved in ethylene carbonate (EC), dimethyl carbonate, and ethyl methyl carbonate]. Li–Cu cells were assembled to evaluate the feasibility of employing the 3D-COF separator for Li metal anodes (Fig. S20). At a current density of 1 mA cm^−2^, the cell with the 3D-COF separator exhibits a higher coulombic efficiency (CE) than that with the PP separator. These results demonstrate that the 3D-COF separator enables more reversible Li deposition and stripping at the anode. The 3D-COF and PP separators were each soaked with electrolyte and then sandwiched between 2 Li-metal sheets to assemble Li–Li batteries. During the initial few cycles (Fig. 3B), at a current density of 1 mA cm^−2^ (areal capacity of 1 mAh cm^−2^), the optimized 3D-COF separator showed significantly reduced voltage polarization (approximately 10 mV), compared to about 80 mV for the PP membrane. After cycling for 500 h, the voltage polarization of the 3D-COF separator remained relatively stable, while the PP membrane exhibited significant voltage polarization. The battery with the optimized 3D-COF separator could cycle for over 1,000 h, far exceeding the cycle life of the battery with the PP membrane. This demonstrates that the COF membrane can significantly improve the cycling performance of the battery and mitigate battery aging. Furthermore, Li–Li cells were assembled to determine the Li-ion transference number using the Bruce–Vincent method. As shown in Fig. S22, the Li-ion transference number of the PP separator was 0.43, whereas that of the 3D-COF separator was significantly enhanced to 0.72. In addition, a composite separator (3D-OH COF powder@PP) was fabricated by coating 3D-OH COF powder onto a PP substrate, and its electrochemical performance in Li–Li batteries was evaluated under identical conditions (Fig. S22). The results show that its polarization voltage is lower than that of the pristine PP separator but higher than that of the 3D-COF membrane, indicating that the COF membrane prepared via in situ interfacial polymerization is more favorable for facilitating rapid ion transport.

Notably, the PP cell containing commercial (commonly used) lithium-ion battery electrolyte exhibited significantly increased cell polarization (about 400 mV) after 220th cycles and showed clear deterioration, with a short circuit occurring at the 250th cycle due to easily recognizable Li^0^ dendrite growth (Fig. 3C, right). A thick black layer was also visibly observed on the surface of the Li^0^ sheet (Fig. 3C, right inset). In contrast, the Li^0^ sheet in the 3D-COF cell exhibited a certain metallic luster (Fig. 3C, left inset), strongly indicating the absence of parasitic reactions or Li^0^ dendrites on the Li-metal electrode (Fig. 3C, left). To further elucidate the interfacial reactions, x-ray photoelectron spectroscopy (XPS) chemical analysis was performed with a sputtering time of 300 s, corresponding to a depth of approximately 150 nm (Fig. S23). In cells with PP separators, stronger C–C and C–H signals were detected, which can be attributed to the decomposition of organic molecules in the electrolyte and subsequent deposition of byproducts on the electrode surface. In contrast, cells employing the 3D-COF separator exhibited a more pronounced LiF signal, which favors the formation of a more robust and compact solid electrolyte interphase (SEI) layer. The XPS depth profile further revealed that the amount of electrolyte decomposition byproducts was significantly lower in the 3D-COF cell, indicating that the 3D-COF separator promotes the formation of a denser and thinner SEI layer, thereby suppressing continuous parasitic reactions between the electrolyte and Li metal and enabling more uniform Li deposition.

Electrochemical impedance spectroscopy (EIS) (Fig. S24) showed that, after 15 cycles, the battery based on the 3D-COF separator exhibited significantly lower SEI resistance compared to the battery with the original PP separator. Additionally, even under extreme conditions of high current density (10 mA cm^−2^, areal capacity of 10 mAh cm^−2^), the 3D-COF cell demonstrated very low polarization voltage (70 mV) (Fig. S25), significantly outperforming the battery with the PP separator. This also confirms that the uniform nanoporous 3D-COF membrane can suppress Li^0^ dendrite formation. This is closely related to the ability of 3D-COF to promote Li^+^ desolvation, thereby inhibiting continuous parasitic reactions between the Li-metal electrode and the liquid electrolyte.

More importantly, in high-voltage Li-metal batteries (Li|NMC622 full cells) using a high-energy LiNi_0.6_Mn_0.2_Co_0.2_O_2_ cathode, Li metal anode, the 3D-COF separator performed well under actual battery conditions (Fig. 3D). The cell with the 3D-COF separator were cycled at a 1-C rate within a charge–discharge voltage range of 2.7 to 4.6 V. Over 300 cycles, the capacity degradation of the cell with the original PP separator was significantly greater than that of the battery with the 3D-COF separator, and the latter also exhibited higher CE. Impressively, after 300 cycles, the Li^0^ surface in the battery with the 3D-COF separator showed no obvious dendritic growth (Fig. 3E, left), in sharp contrast to the disordered Li^0^ surface observed in the cell with the PP separator (Fig. 3E, right). Electrochemically, the cell with the 3D-COF separator also outperformed the one with the PP separator, with a capacity retention rate of 81.4% after 300 cycles, compared to 38.0% for the PP-based battery (Fig. S26). To evaluate the structural stability of 3D-COF, Li|NMC622 full cells were disassembled after 300 cycles, and the 3D-COF membrane was retrieved for XRD and N2 adsorption–desorption measurement (Fig. S27). The results reveal that, although the diffraction peak intensities of the cycled 3D-COF and surface area decreased slightly, the overall diffraction features were well preserved, demonstrating its excellent structural stability during long-term cycling.

### The mechanism of Li^+^-desolvation

**Fig. 4.**
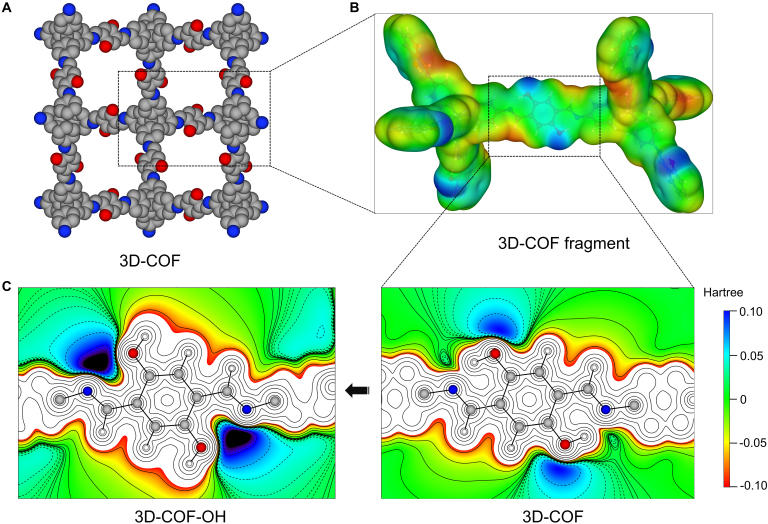
Density functional theory calculated electrostatic potential (ESP) of 3D-COF. (A) Structure of 3D-COF (carbon, gray; oxygen, red; nitrogen, blue). (B) ESP of 3D-COF fragment. (C) 2D section of ESP (in the C–OH plane).

In order to further explore the mechanism by which 3D-COF stabilizes the lithium metal anode, we employed density functional theory (DFT) calculations to investigate the electronic structure of the COF and its interactions with lithium-solvated species. Since COF materials are used in the liquid phase of electrolytes, we simplified the infinite structure of the COF to a molecular fragment as shown in Fig. 4B, introducing an implicit solvent field model to represent the electrolyte environment. According to the results of the DFT calculations, the negatively charged sites on the framework, which are capable of interacting with lithium-ion solvation species, are located near the oxygen of the hydroxyl group. To further elucidate the electronic structural characteristics around this fragment, we performed a detailed analysis of the electrostatic potential (ESP) using a 2D cross-sectional approach. As shown in Fig. 4C, it is clearly observed that a significant negative charge (due to the lone pair electrons on the oxygen) is present near the oxygen atom. The lone pair electrons on the nitrogen of the imine group form a hydrogen bond with the hydrogen in the hydroxyl group, exhibiting an overall neutral character. However, when the hydrogen on the hydroxyl group undergoes a conformational flip, a very prominent electron-rich region forms between oxygen and nitrogen (3D-COF-OH).

**Fig. 5.**
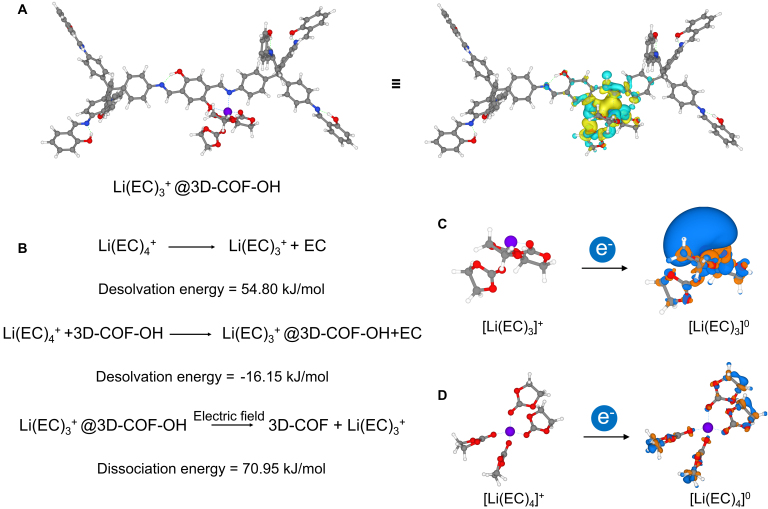
Density functional theory calculated energy profile for the reduction process of Li^+^-solvates. (A) Charge density difference (CDD) of the binding process of Li(EC)_3_^+^ to 3D-COF-OH. (B) Desolvation energy under free electrolyte and in 3D-COF. CDD in the electro-reduction process of Li(EC)_3_^+^ (C) and Li(EC)_4_^+^ (D) (carbon, gray; oxygen, red; hydrogen, white; nitrogen, blue; Li, purple).

Upon the approach of lithium-ion solvated species, lithium can form strong interactions with the oxygen and nitrogen in this region. As depicted in Fig. S28 (above line), the distances between Li and O and between Li and N are 1.995 and 2.170 Å, respectively. In contrast, the distances between Li^+^ and 3 other solvent molecules, EC, are 2.271, 1.975, and 1.970 Å. Notably, EC, as a cyclic carbonate molecule, is highly polar, and its carbonyl oxygen forms a strong solvation bond with Li^+^. This oversolvation has been identified in previous studies as one of the primary triggers for side reactions at the lithium metal anode surface [48–52]. To further investigate the interaction between lithium solvation species and the electron-rich sites of 3D-COF-OH, we calculated the charge density difference (CDD) during their binding process. As shown in Fig. 5A (Fig. S28, zoomed-in image), the electrons around the O and N sites on the COF framework notably flow toward Li^+^, with increased electron density between O...Li and N...Li (the yellow area represents regions where electrons are enriched), indicating a strong interaction between the electron-rich O and N sites of 3D-COF-OH and lithium-solvated species. Meanwhile, the electron density around the carbonyl oxygen of EC (directly coordinated to Li^+^) decreases (the cyan area represents regions where electrons are depleted), and these electrons are transferred to the carbon sites of the EC molecule. This CDD suggests that the interaction between 3D-COF-OH and lithium-solvated species mitigates the strong polarization of the EC molecule by Li^+^, potentially reducing the tendency for solvent reduction side reactions.

This strong interaction between lithium-solvated species and the electron-rich sites of the COF framework is likely the main reason why the 3D-COF membrane demonstrates effective suppression of side reactions at the lithium metal surface. The strong inductive effect of Li^+^ on electrolyte solvent molecules leads to the polarization of solvent molecules, imparting partial positive charges to their surfaces [Fig. S29, the charge distribution of Li(EC)_4_^+^]. Conversely, the coordination-saturated lithium solvation species, upon receiving electrons during the reduction process (blue area in Fig. 5D), will predominantly have their electron density located at the methylene positions of the EC molecule, rather than around Li^+^, which could trigger solvent reduction reactions (side reactions on the lithium metal anode surface). The partial desolvation of coordination-saturated lithium ions, such as via the SEI layer on the anode surface [53], is considered a key factor in promoting the normal electrochemical reduction of Li^+^ and achieving stable cycling of the anode material [54]. The SEI layer that assists in the partial desolvation of Li^+^ is the underlying mechanism for the stable operation of graphite anodes in lithium-ion batteries [55], enabling rechargeable batteries to cycle stably over long periods. However, lithium metal, as a hostless anode material, experiences significant volumetric changes during charge/discharge cycles. Coupled with the rigid and brittle nature of the SEI layer [56,57], which causes it to continuously fracture and regenerate during cycling (mainly due to solvent reduction side reactions), it becomes a major obstacle to the stable cycling of lithium metal anodes.

From an energy perspective, as calculated using the implicit solvent field model in DFT, the involvement of 3D-COF-OH promotes the desolvation of Li(EC)_4_^+^ due to the strong interactions between the electron-rich N and O sites on the framework and Li^+^. In free electrolyte solutions, fully solvated species like Li(EC)_4_^+^ require an energy of approximately 54.80 kJ/mol to shed one solvent molecule and form Li(EC)_3_^+^. However, the involvement of 3D-COF-OH reduces this energy to −16.15 kJ/mol. On the other hand, during the charging process of the battery, lithium solvation species will move toward the anode under the influence of the electric field. The Li(EC)_3_^+^ @ 3D-COF-OH complex formed by the COF, due to the immobile nature of the framework, will undergo further dissociation under the electric field, producing free Li(EC)_3_^+^. The dissociated Li(EC)_3_^+^, if it undergoes reduction, will primarily gain electrons at the Li^+^ sites. Of course, this Li(EC)_3_^+^ is coordination-unsaturated, and after dissociating from the COF, it can progressively become fully coordinated in the electrolyte. However, since our 3D-COF is in direct contact with the lithium metal and has a very smooth surface (Fig. 2B), which reduces the distance between the COF material and the lithium metal anode, partially desolvated species formed inside the COF pores may undergo electrochemical deposition before fully coordinating [48], leading to significant lithium anode stabilization effects in lithium-metal batteries equipped with 3D-COF membranes (Fig. 3C and E).

## Conclusion

In summary, a large-area, uniform, and continuous nanoporous 3D-COF membrane was fabricated via a substrate-assisted interfacial in situ polymerization strategy. The interconnected 3D nanochannels within the 3D-COF membrane facilitate the smooth passage of Li^+^ and electrolytes, while the solvation cages formed by hydroxyl and imine groups on the COF framework inhibited Li-dendrites and parasitic reactions at the Li-metal electrode. The Li^+^-solvation cage and the dense nanoporous feature of the 3D-COF membrane demonstrated in this work may provide inspiration for this long-standing challenge in the battery field. Moreover, we present a simple method for fabricating large-sized, uniform, and continuous COF membranes, overcoming the challenge of porous material membrane formation, and holding promise for advancing the widespread application of COF materials.

## Methods

Preparation of 3D-COF membrane: TAM (380 mg, 1 mmol) was dissolved in water (600 ml) and then acetic acid (6 M, 1 ml) was added to prepare an aqueous solution. Then, the aqueous phase was added to the bottom of a flat-bottomed glass dish, and a porous PP membrane was placed on top of the aqueous phase. DHTA (332 mg, 2 mmol) was dissolved in n-octanoic (600 ml) and then layered on top of the aqueous solution. The organic aqueous system remained undisturbed at room temperature (25 °C) for 15 d. The membrane was then washed with tetrahydrofuran (THF) 10 times and dried at 60 °C for 24 h to obtain the separator, named 3D-COF.

## Data Availability

All data needed to support the conclusions in the paper are presented in the manuscript and the Supplementary Materials. Additional data related to this paper may be requested from the corresponding author upon request.
