# Supplementary material for: A Large-Area Uniform Three-Dimensional Covalent Organic Framework Membrane for Stabilizing Li-Metal Electrodes via Solvation Cages
Source: Research (Wash D C). 2025 Oct 9;8:0926. doi: 10.34133/research.0926 (PMC12508527; doi:10.34133/research.0926)
Supplement: Supplementary 1 — Supplementary Text Figs. S1 to S29 Tables S1 to S6 [file research.0926.f1.pdf]

Supporting Information

A large-area uniform three-dimensional covalent organic framework membrane for stabilizing Li-metal electrodes via solvation cages

*Zhuozhuo Tang[‡], Jia Chen[‡], Da Zhu, Li Sheng, Yang Yang, Kai Yang, Jianlong Wang, Yaping Tang, Xiangming He, and Hong Xu**

*Corresponding authors: hongxu@tsinghua.edu.cn

[‡] These authors contributed equally.

This PDF file includes:

Supplementary Text

Figs. S1 to S29

Tables S1 to S6

References

Supplementary Methods

Materials

Unless otherwise specified, all starting materials and solvents were procured from J&K Scientific LTD. and used as received without further purification. All products were isolated and handled under a nitrogen atmosphere, utilizing glovebox or Schlenk line techniques.

Preparation of 3D-F-COF membrane

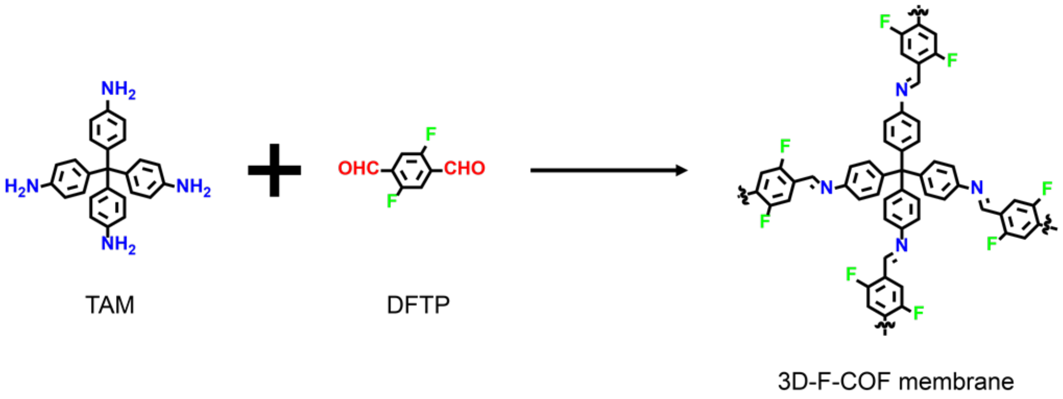

Tetra-(4-anilyl)-methane (TAM, 38 mg, 0.1 mmol) was dissolved in water (60 mL) and then added acetic acid (6 M, 0.1 mL) to prepare an aqueous solution. Then, the aqueous phase was added to the bottom of a flat-bottomed glass dish, and a porous polypropylene (PP) membrane was placed on top of the aqueous phase. 2,5-difluoro-1,4-benzenedicarboxaldehyde (DFTP, 34mg, 0.2 mmol) was dissolved in n-octanoic (60 mL) and then layered on top of the aqueous solution. The organic-aqueous system remained undisturbed at room temperature (25 oC) for 15 days. The membrane was then washed with THF 10 times, and dried at 60 oC for 24 hours obtained 3D-F-COF membrane.

Synthesis of 3D-COF powder

3D-COF powder was synthesized following a modified version of a previously reported method.[1] In a 20 ml Pyrex tube, 38 mg (0.1 mmol) of tetra-(4-anilyl)-methane (TAM) and 33.2 mg (0.2 mmol) of 2,5-dihydroxyterephthalaldehyde (DHTA) were combined with 0.5 ml of anhydrous 1,3,5-trimethylbenzene and 0.5 ml of o-dichlorobenzene. The

mixture was sonicated until fully dispersed, after which 0.1 ml of 6M acetic acid were added. The tube was flash frozen at 77 K (LN_2 bath), evacuated to an internal pressure of ~ 0.1 MPa, and flame sealed. The reaction mixture was then heated to 120 °C for three days. The resulting orange powder was collected by centrifugation and thoroughly washed using a Soxhlet extractor with THF for 24 hours. Finally, the sample was dried in vacuum at 120 °C for 24 hours, yielding a yellow 3D COF powder.

Preparation of 3D-OH COF powder@PP

The composite 3D-OH COF powder@PP separator was fabricated by a slurry-coating method. 3D-OH COF powder and PVDF were dispersed in NMP to make a slurry with a ratio of 6:4 by weight at room temperature. Then the slurry was stirred for 0.5 h and coated onto a commercially available 25 μm microporous polyethylene separator (PP, Celgard 2500) with a doctor blade. The composite separator was dried in a drying oven at 70 °C for 3 h, and vacuum oven at 70 °C for 12 h.

Characterization methods

The stress-strain curves were measured using a Shimadzu AGS-X universal mechanical drawing machine. Powder X-ray diffraction (PXRD) data were collected using a Bruker AXS D8 DISCOVER X-ray diffractometer (λ = 1.5418 Å, 40 kV, 40 mA). Samples were deposited on a glass substrate and scanned from 2θ = 2° to 40° in 0.02° increments. Solid-state ^{13}C NMR spectra were acquired on a JEOL JNM-ECZ600R 600 MHz spectrometer. Fourier transform infrared (FT-IR) spectra were recorded using a JASCO FT-IR-6100 spectrometer with KBr pellets. Thermogravimetric analysis (TGA) was conducted under nitrogen using a METTLER TOLEDO 1600LF thermal analyzer, with a heating rate of 30 °C min^{-1} up to 800 °C and a nitrogen flow rate of 10 mL min^{-1}. Transmission electron microscopy (TEM) was performed with a JEM-2100 electron microscope (Japan) at an accelerating voltage of 200 kV. Scanning electron microscopy (SEM) images were obtained using a Zeiss Gemini microscope. Elemental distributions were analyzed through energy-dispersive X-ray spectroscopy (EDS) mapping with an energy-dispersive detector in the SEM. The sorption isotherm for N_2 was measured by

using a BEL sorp-max Ⅱ analyzer with ultra-high-purity N_2 (99.999% purity). X-ray photoelectron spectroscopy (XPS) measurements were carried out on ESCALAB Xi+, which used Al Ka radiation (15 kV, 10 mA) and an argon ion beam; the pressure was lower than 10^{-9} torr. Depth profiling was fulfilled using argon ion sputtering at the rate of 30 nm per minute for Si.

Measurements

The galvanostatic charge/discharge evaluations were conducted at ambient temperature using a CT2001A cell testing device from LAND Electronic Co. Ltd for cells with liquid electrolytes.[2, 3] Li | Li symmetric cells containing liquid electrolyte were examined at a current density of 1.0 mA cm^{-2}.[4, 5] Battery assembly was conducted in an argon-filled glove box. To ensure experimental consistency, 60 μl of liquid electrolyte consisting of 1.0 M $LiPF_6$ in EC/DMC/EMC (1:1:1 by weight) with 1 wt% VC was used.[6, 7] The cells were initially fully charged prior to observation and subsequently discharged at fixed current densities of 1.0 mA cm^{-2} and 10.0 mA cm^{-2}.

The cathodes were composed of $LiNi_{0.6}Mn_{0.2}Co_{0.2}O_2$ (NMC622) active material, Super P, and PVDF in an 8:1:1 mass ratio, with NMP as the dispersant [8, 9] The NMC622 electrode sheets had an active material weight density of 4.5 mg cm^{-2} and an areal capacity loading of 0.855 mAh cm^{-2}. The cathodes, 12 mm in diameter, were paired with lithium metal foil anodes, approximately 400 μm thick and 15.4 mm in diameter, used as received without treatment. The cell stack pressure was 50 kg cm^{-2}, and the charge/discharge voltage range was 2.7-4.6 V. Formation cycles were conducted at 0.1 C for two cycles, followed by cycling at 1 C.

The impedance studies of Li-symmetric cells with PP separators and 3D-COF@PP were executed using a CHI660E Electrochemical Workstation (Shanghai Chenhua) through electrochemical impedance spectroscopy (EIS). [10, 11] The measurements were performed with a perturbation amplitude of 10 mV, covering a frequency range from 0.1 Hz to 10 kHz at ambient temperature.

The Li^+ transference number (t_{Li+}) was calculated according to theBruce-Vincent-Evans equation:

$$t_{Li^+} = \frac{I_s(\triangle V - I_0 R_0)}{I_0(\triangle V - I_s R_s)}$$

where ΔV was the polarization potential (10 mV), I_0 and R_0 were the initial current and initial interfacial resistances, I_s and R_s were the steady-state and steady-state interfacial resistances after potential polarization.

Theoretical calculations

The crystal structure of 3D-COF was using the density functional theory (DFT) [12, 13] implemented in the Vienna Ab Initio Simulation Package (VASP)[14, 15], with the generalized gradient approximation (GGA) using Perdew–Burke–Ernzerhof (PBE)[16] as functional for exchange and correlation potential and projector augmented wave pseudopotentials (PAW)[17] with cut-off energy of 500 eV. Grimme dispersion correction[18, 19] with Becke-Johnson damping function[20] was employed to describe van der Waals (vdW) interactions. Both atomic coordinates and lattice dimensions were optimized in the calculation. To include the contribution of the electrolyte environment, the VASP-optimized COF structure was further constructed into molecular fragment (Fig. 4b); and together with other Li^+-solvates were then calculated with an implicit solvent model using using SMD solvation model[21] in Gaussian 16 package[22]. The calculation was performed at PBE0[16, 23, 24]/def2-SVP [25, 26] level of theory; acetone parameters were used to represent the commonly used electrolyte in the SMD models[27]. Multiwfn[28] was employed to analyze the electronic structures of Gaussian 16 calculation results; while Discovery Studio Visualizer[29] and VESTA[30] were employed to visualize crystal/molecular structures and electronic structures.

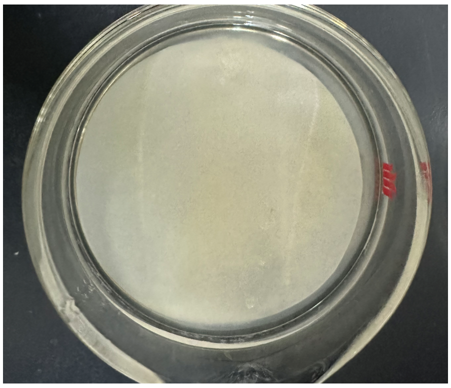

Fig. S1. Photograph of 3D-F-COF membrane.

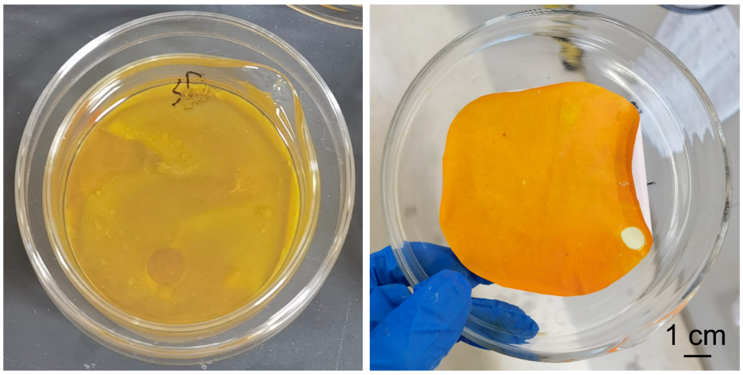

Fig. S2. Optical photograph of the 3D-COF@PP membrane.

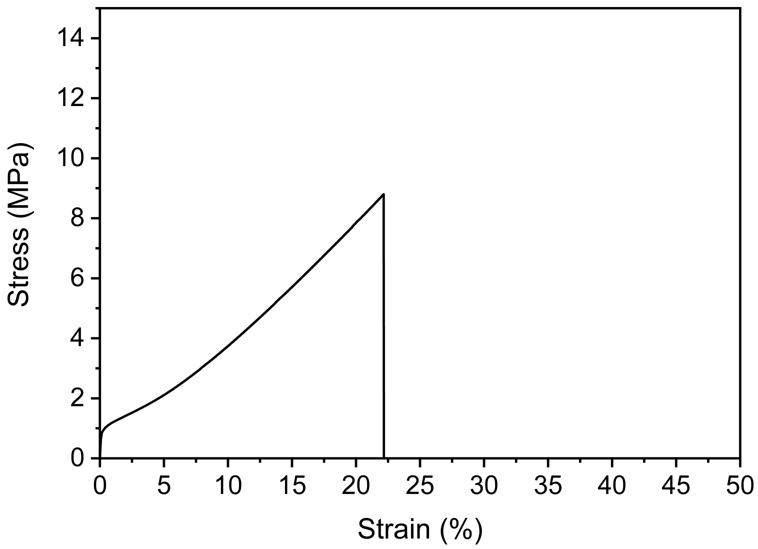

Fig. S3. The stress-strain curve of the 3D-COF membrane.

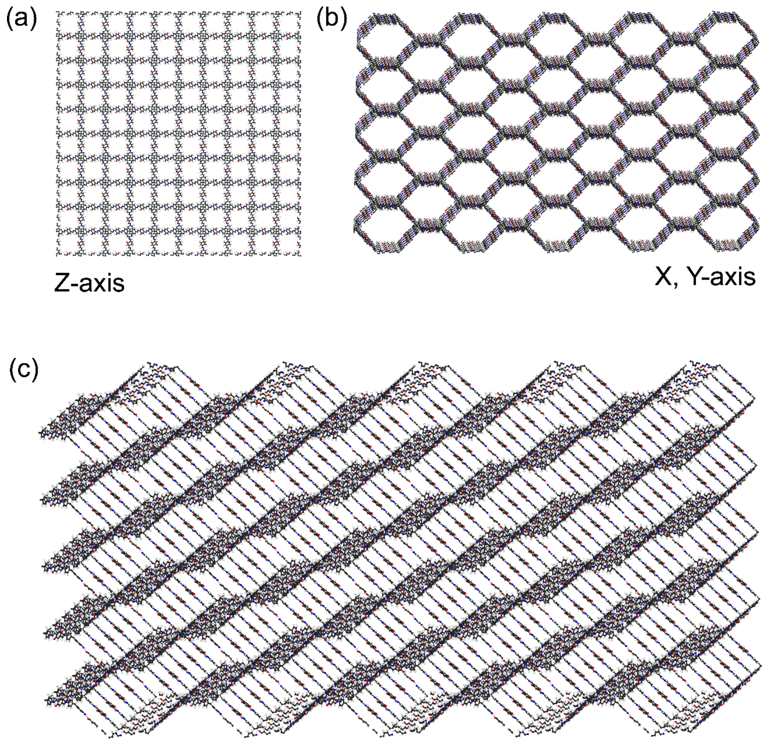

Fig. S4. Periodic stacking structures of non-interpenetrating 3D-COF membrane in different orientations.

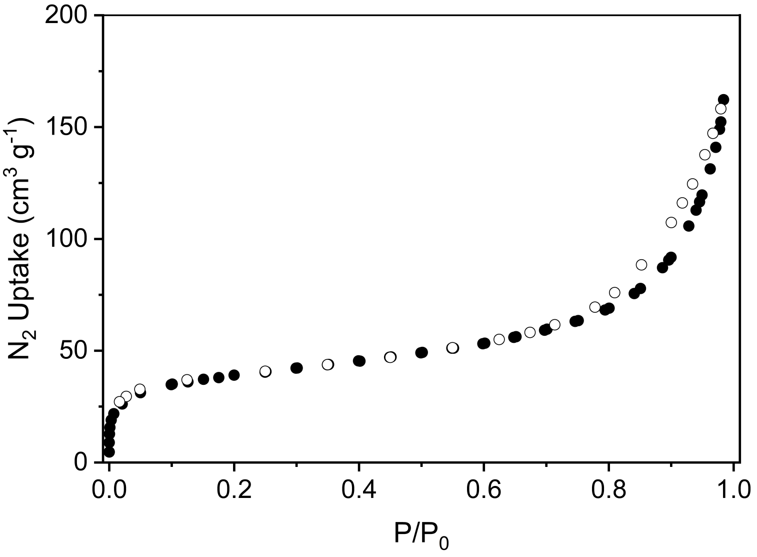

Fig. S5. N_2 adsorption-desorption isotherm of 3D-COF membrane.

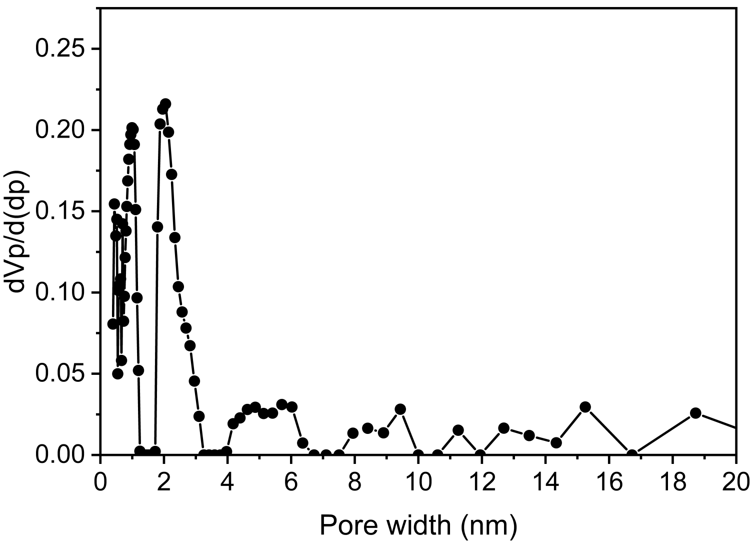

Fig. S6. Pore size distribution of 3D-COF membrane.

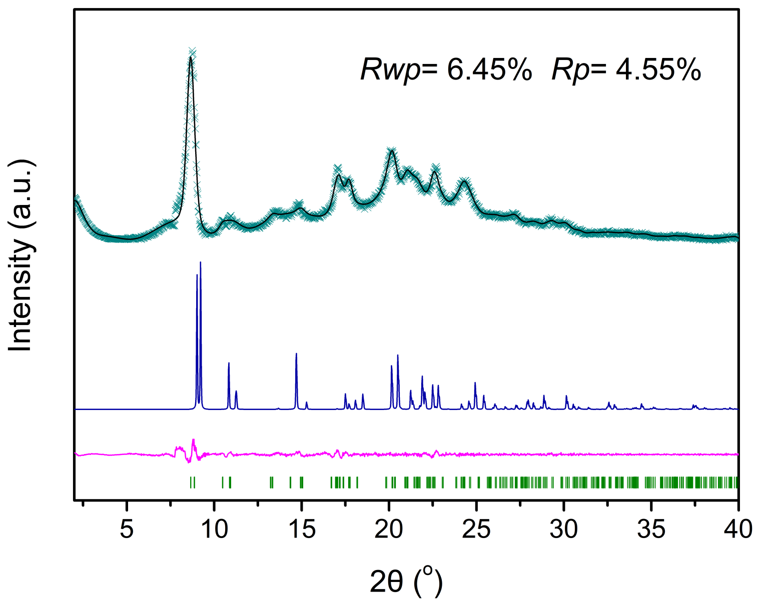

Fig. S7. XRD pattern of 3D-COF powder (XRD refinement, experimental pattern: light blue, Pawley refined pattern: black curve, calculated pattern from the non-fold dia model: blue curve, difference pattern: purple curve, Bragg positions: green).

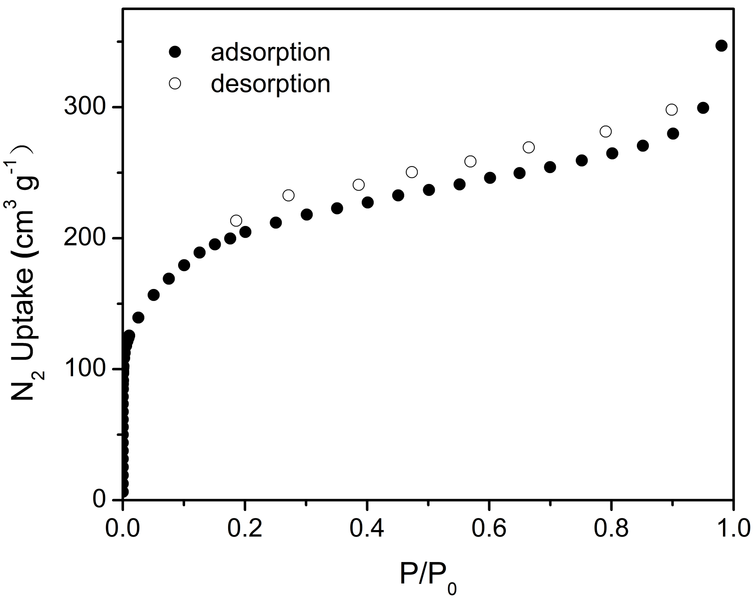

Fig. S8. N_2 adsorption-desorption isotherm of 3D-COF powder

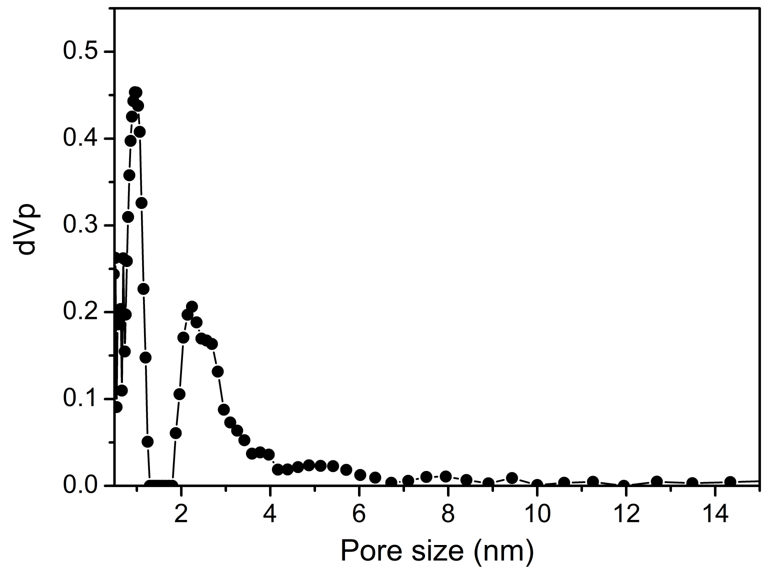

Fig. S9. Pore size distribution of 3D-COF powder calculated from N_2 adsorption isotherm at 77 K.

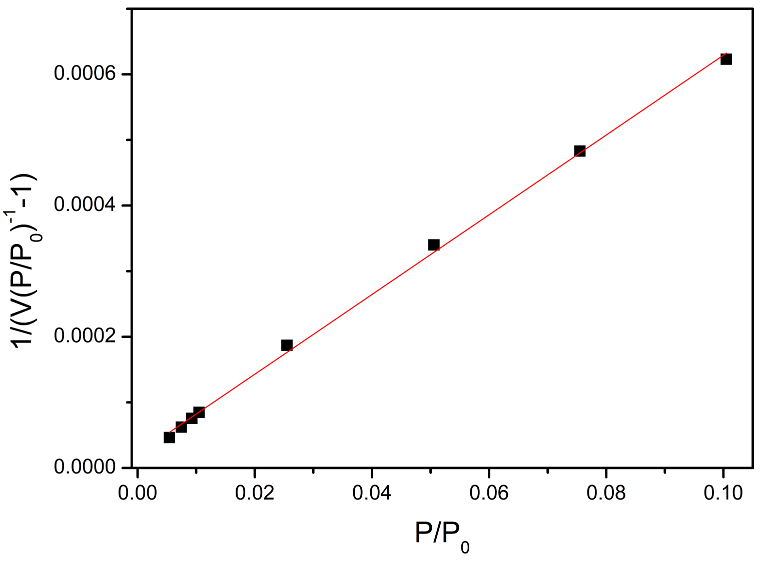

Fig. S10. BET plot of 3D-COF powder calculated from N_2 adsorption isotherm at 77 K.

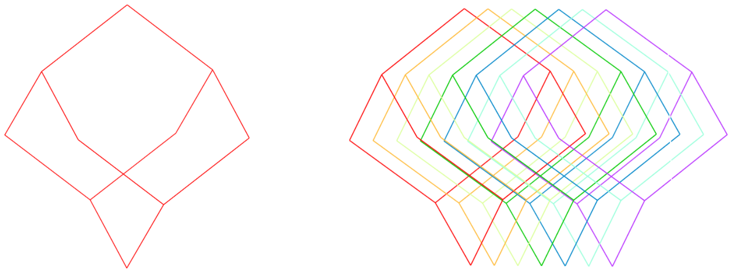

3D-COF-(non-fold-dia) 3D-COF-(7-fold-dia)

Fig. S11. Schematic representation of different interspersed dia topologies: 3D-COF membrane (non-fold-dia) and 3D-COF powder (7-fold-dia).

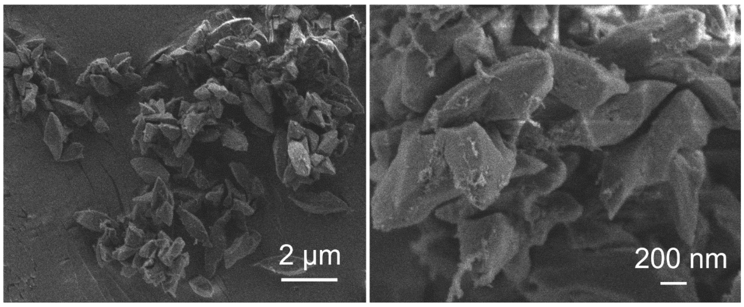

Fig. S12. SEM images of 3D-COF powder.

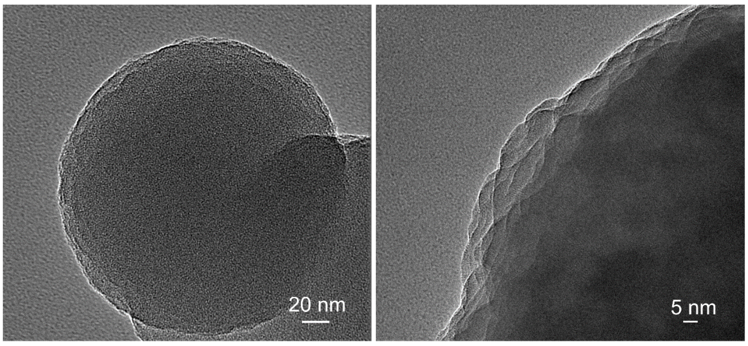

Fig. S13. TEM images of 3D-COF membrane.

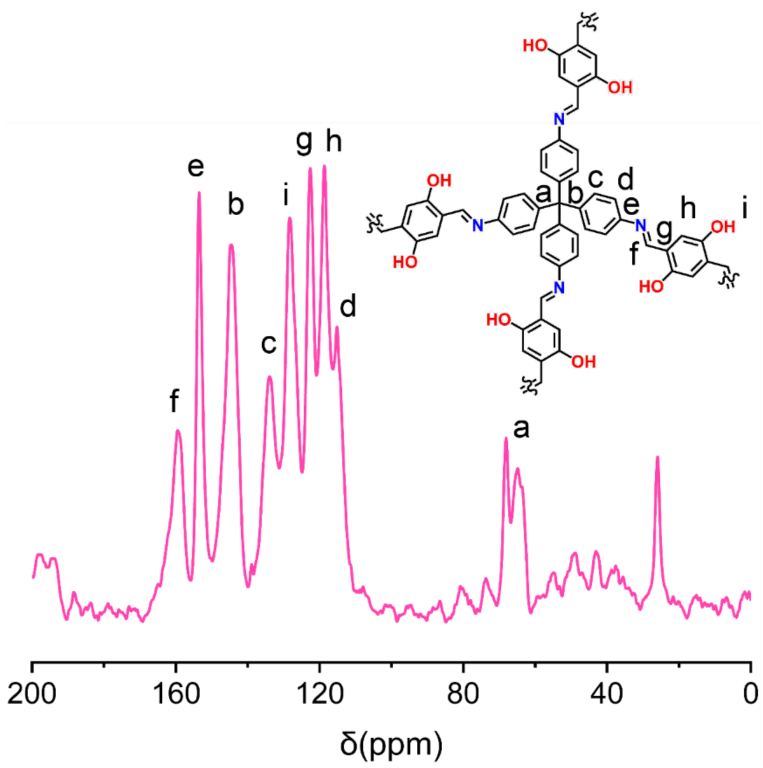

Fig. S14. ^{13}C NMR spectrum of 3D-COF membrane.

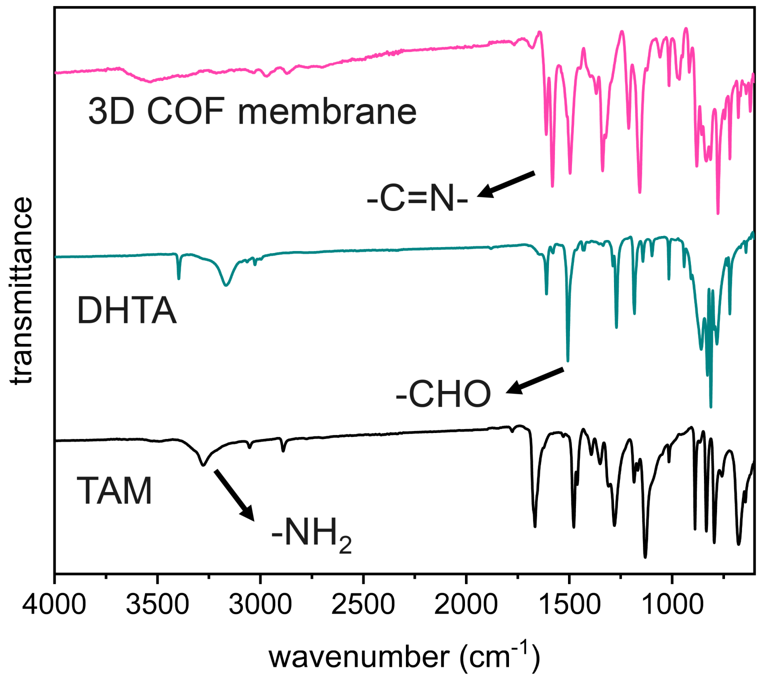

Fig. S15. FTIR patterns of 3D-COF membrane and monomers.

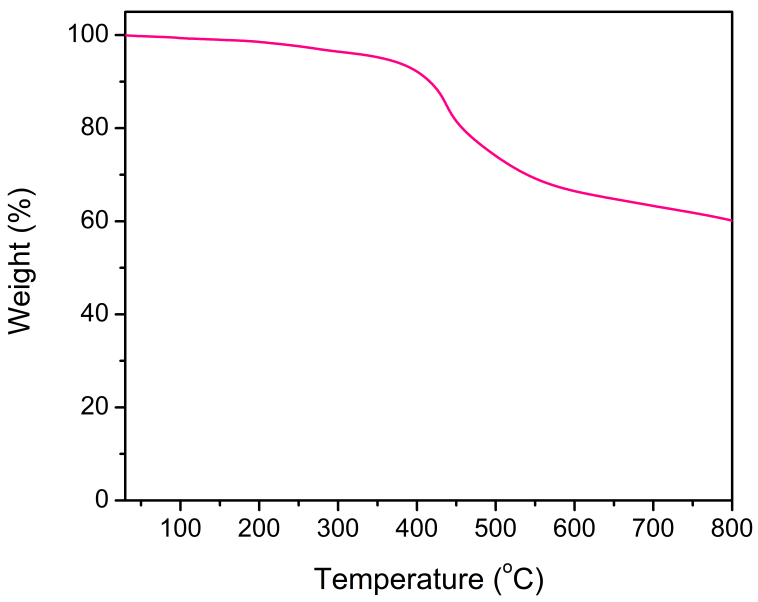

Fig. S16. TGA curve of 3D-COF membrane.

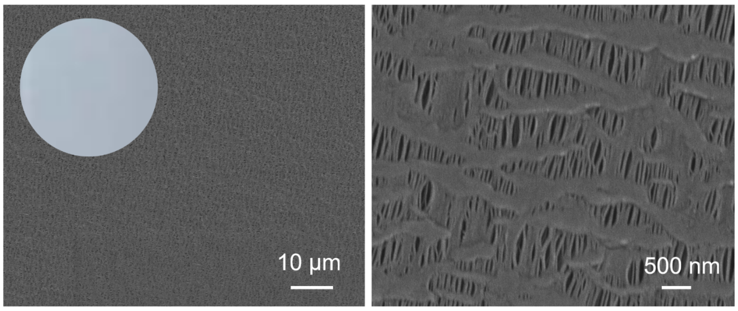

Fig. S17. SEM image of 3D-COF@PP membrane. left: (PP side, inset: optical picture of PP side), right: cross section of PP membrane.

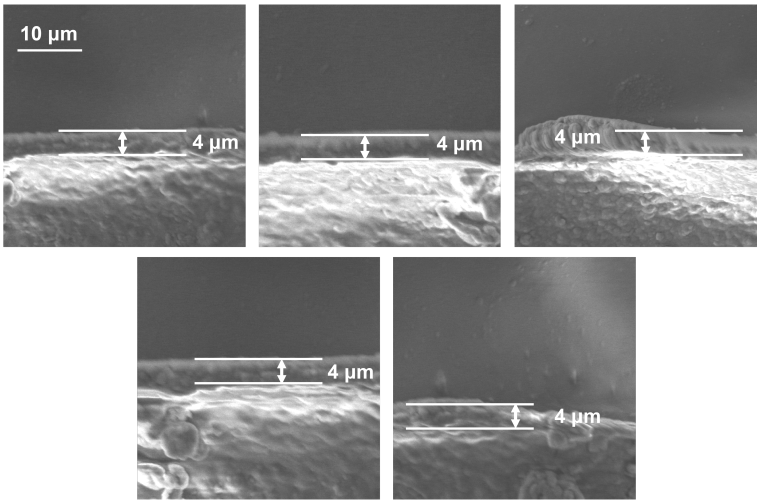

Fig. S18. Cross-sectional SEM images of the large-area 3D-COF membrane collected from five uniformly selected positions.

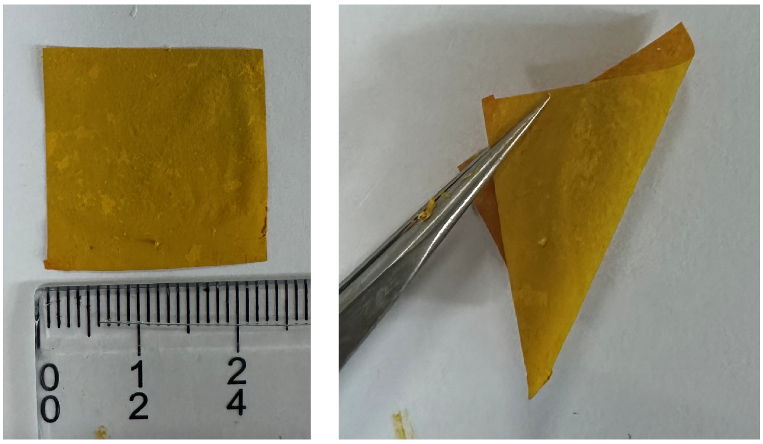

Fig. S19. Optical photograph of self-standing 3D-COF membrane.

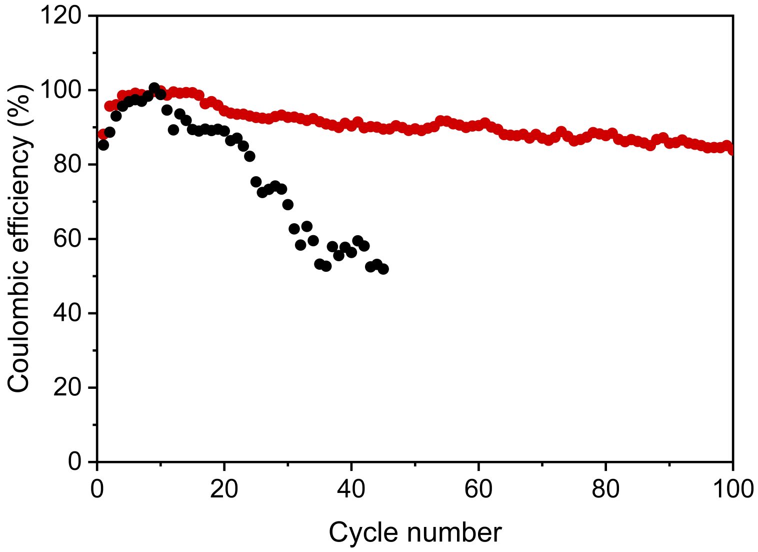

Fig. S20. Comparison of the Coulombic efficiency curves of Li-Cu cells with PP (black) and 3D COF (red) separators at a current density of 1 mA cm^{-2}.

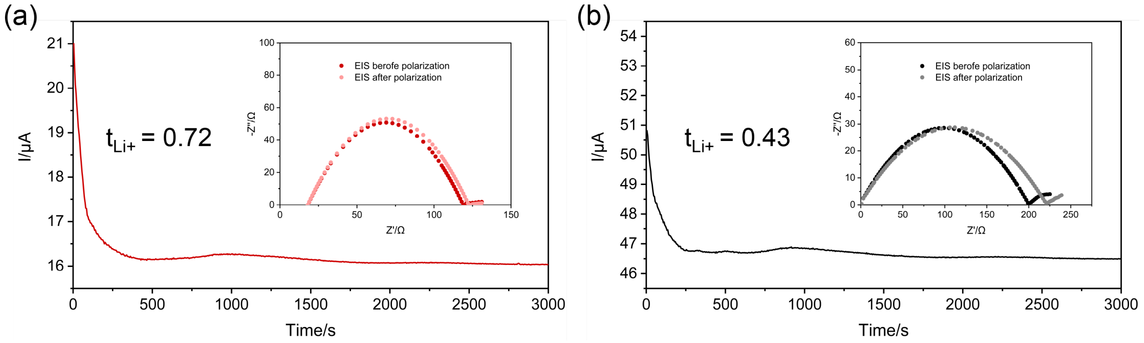

Fig. S21. The steady-state polarization curve of the lithium symmetric battery assembled by 3D COF (a) and PP (b) separator (insets: the EIS curve before and after polarization).

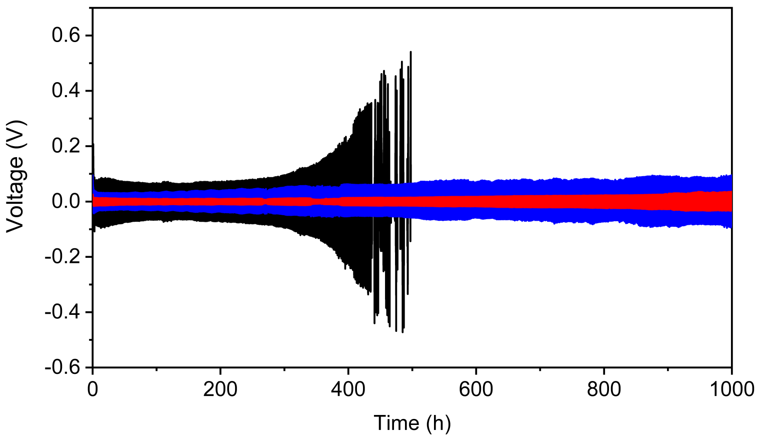

Fig. S22. Initial voltage profiles of the PP separator (black), 3D-OH COF powder@PP (blue), and 3D-COF membrane (red)-based cells at a fixed current density of 1 mA cm^{-2}.

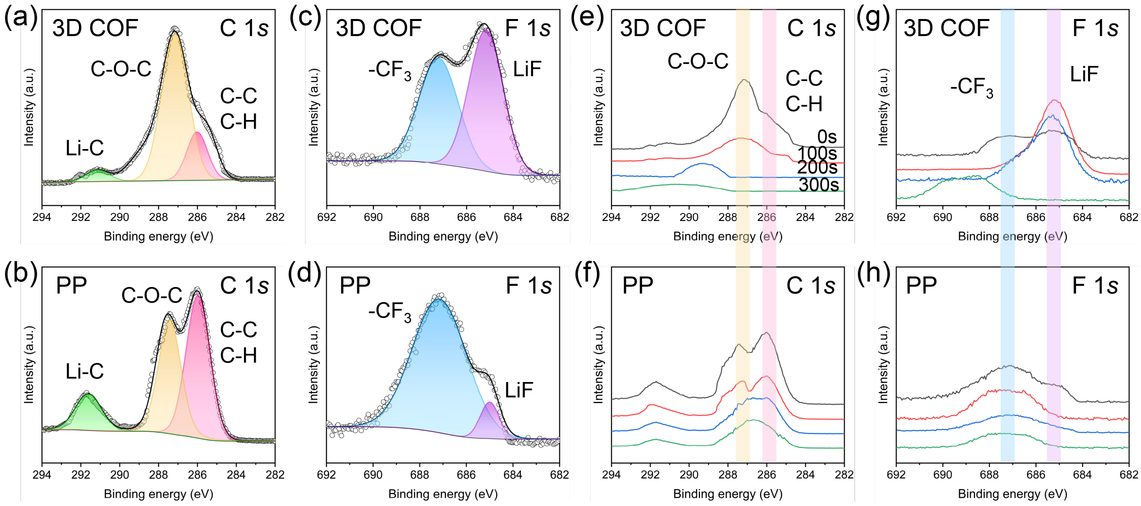

Fig. S23. (a, b, c, d) Detailed XPS spectra of carbon (C 1s (a and b)) fluorine (F 1s (c and d)), on the cycled Li anode in the 3D-COF and PP separator-based cells, respectively; (e, f, g, h) the corresponding spectra of F and C elements at cycled Li anode surface were recorded at different times.

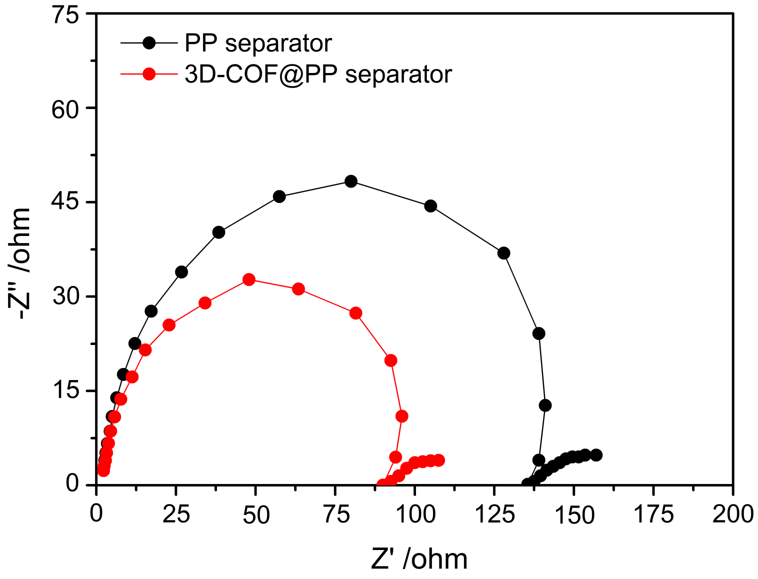

Fig. S24. Impedance spectroscopy of Li-symmetric cells with the PP separator and 3D-COF@PP after 10th stripping/plating at a current density of 1 mA cm^{-2}.

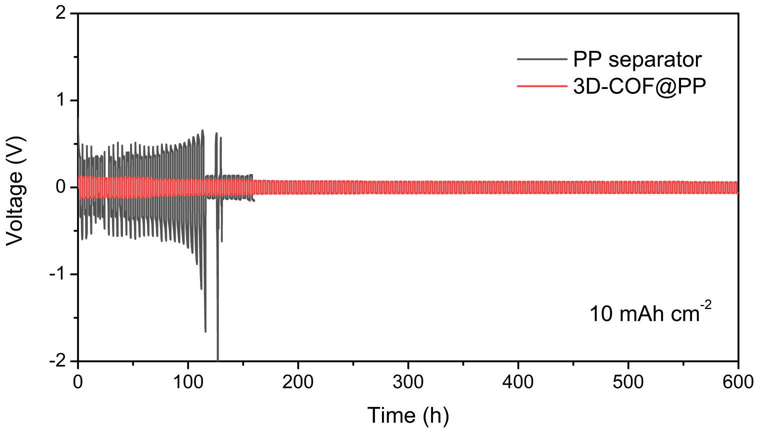

Fig. S25. Initial voltage profiles of the PP separator (black) and 3D-COF@PP (red)-based cells at a fixed current density of 10 mA cm^{-2}.

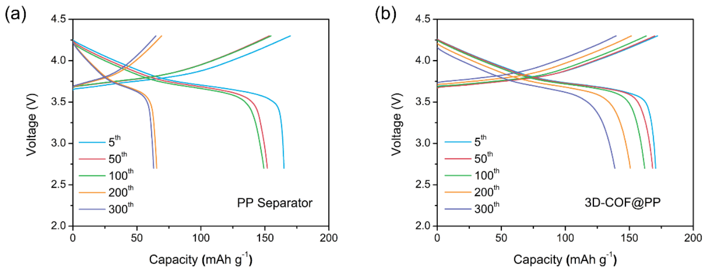

Fig. S26. Charge/discharge voltage profiles with the PP separator (a) and 3D-COF@PP-based (b) cells.

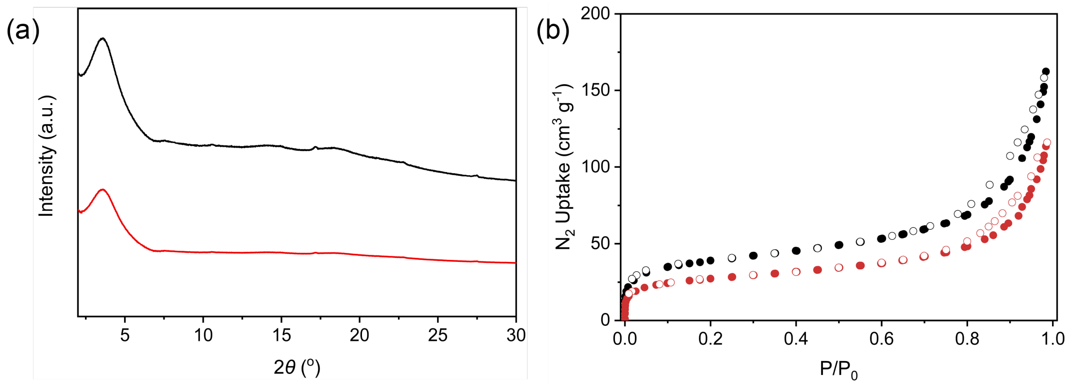

Fig. S27. (a) XRD patterns of the pristine 3D-COF (black) and the 3D-COF after 300 cycles in the Li | NMC622 full cell (red). (b) N_2 adsorption–desorption isotherm of the pristine 3D-COF (black) and the 3D-COF after 300 cycles in the Li | NMC622 full cell (red).

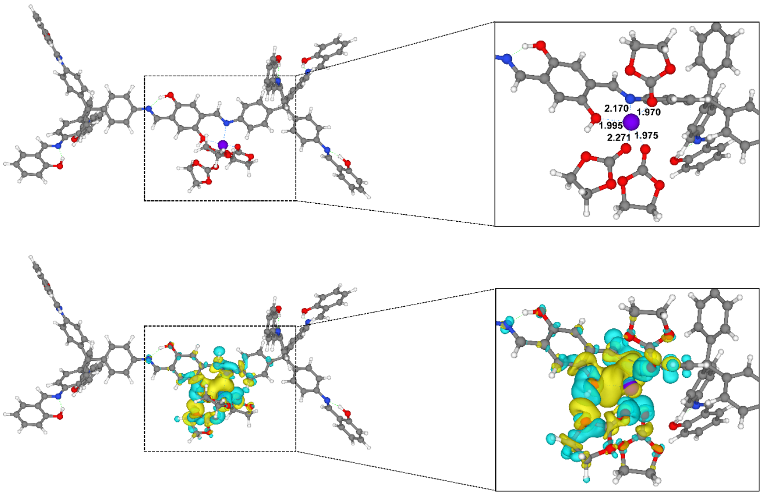

Fig.S28. A localized magnified view of the CDD for the binding process of $Li(EC)_3^+$ to 3D-COF-OH.

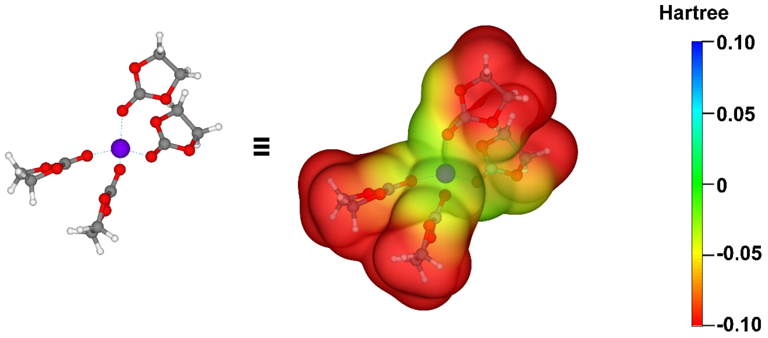

Fig. S29. Electrostatic potential (ESP) of $Li(EC)_4^+$.

Table S1. Atomistic coordinates for the refined unit cell parameters for 3D-COF (non-fold-dia) (space group *I41/A*, $a = b = 26.9474$ Å, $c = 53.1700$ Å, $\alpha = \beta = \gamma = 90°$).

**Atom**	***x*/a**	***y*/b**	***z*/c**
C1	0.5	0.5	0
C2	0.7798	0.45757	0.87605
C3	0.78889	0.49359	0.85738
C4	0.82942	0.48627	0.83982
C5	0.75835	0.53705	0.85638
N6	0.65994	0.48137	0.92756
C7	0.6213	0.48836	0.94497
C8	0.59947	0.4457	0.95549
C9	0.55059	0.56142	0.02683
C10	0.50429	0.54445	0.0183
C11	0.56596	0.53828	0.97099
C12	0.60384	0.53494	0.95317
H13	0.80303	0.42432	0.87697
H14	0.85164	0.45214	0.84228
H15	0.76553	0.57287	0.83898
H16	0.61269	0.40939	0.94927
H17	0.58445	0.54489	0.01922
H18	0.55317	0.57484	0.97707
H19	0.62086	0.5689	0.9459
H20	0.29521	0.56173	0.9215

Table S2. Atomistic coordinates for the refined unit cell parameters for 3D-COF (7-fold-dia) (space group *C2/C*, *a*= c = 20.5286 Å, *c* = 8.5974 Å, *α* = *γ* = 90º, *β* =107º).

Atom	*x*/a	*y*/b	*z*/c
C1	0.76341	0.51124	0.061
C2	0.70746	0.43127	0.07041
C3	0.7168	0.30743	0.11369
C4	0.78251	0.25729	0.1482
C5	0.83872	0.33579	0.14033
C6	0.82955	0.46242	0.09745
C7	0.75133	0.06493	0.21662
C8	0.75452	0.90629	0.23904
C9	0.81254	0.81294	0.24821
C10	0.80661	0.65834	0.25692
C11	0.69063	0.72915	0.99734
C12	0.67675	0.77224	0.05837
C13	0.63525	0.89505	0.05968
C14	0.60761	0.98098	1.00137
C15	0.61934	0.93944	0.93958
C16	0.66043	0.81431	0.93761
C17	0.53452	0.20195	0.96426
C18	0.51287	0.35009	0.98375
C19	0.5172	0.39072	0.05226
C20	0.50603	0.53975	0.06752
N21	0.79267	0.1174	0.18484
N22	0.57547	0.11902	1.01033
O23	0.87752	0.86738	0.25276
O24	0.52943	0.2883	0.10647
H25	0.65347	0.47041	0.04146
H26	0.67089	0.24438	0.12183
H27	0.89232	0.29462	0.16957
H28	0.87558	0.52844	0.09126
H29	0.85195	0.5784	0.26109
H30	0.70074	0.70294	0.10799
H31	0.6234	0.92663	0.11006
H32	0.59497	1.00883	0.89008
H33	0.66954	0.78089	0.8861

H34	0.51489	0.16025	0.90794
H35	0.51234	0.57516	0.12383
H36	0.88991	0.96757	0.28922
H37	0.51326	0.34064	0.14963
C38	1.25	1.12019	0.5

Table S3. Atomistic coordinates of $Li(EC)_3^+$ calculated by using Gaussian 16 with SMD solvation model.

Atom	*x*/(Å)	*y*/(Å)	*z*/(Å)
O	1.163064	-3.21247	-3.05988
O	1.312591	-1.28064	-1.96041
O	2.492037	0.621523	0.400544
O	-0.18292	-2.83351	-1.37033
C	3.187042	-1.05778	3.291025
H	3.531735	-2.09011	3.423661
H	2.439861	-0.81016	4.05901
C	0.799134	-2.3652	-2.11781
C	2.949488	0.192069	1.43546
C	4.316509	-0.04076	3.205503
H	4.430774	0.569232	4.109328
H	5.282687	-0.48963	2.93282
C	0.473698	-4.45865	-2.87005
H	1.1776	-5.17609	-2.4235
H	0.132079	-4.82795	-3.84421
C	-0.6589	-4.06828	-1.9309
H	-1.60458	-3.86646	-2.45485
H	-0.82699	-4.78608	-1.11949
Li	1.261438	0.29652	-0.96575
O	2.564125	-0.9364	2.001826
O	3.892476	0.80434	2.123721
O	0.014188	1.621299	-1.39038
O	0.886986	3.318929	-2.55321
C	-0.10365	2.687447	-1.95123
C	0.369319	4.46544	-3.2473
H	1.039884	5.316073	-3.07757
H	0.328591	4.228796	-4.32038
C	-1.00719	4.635753	-2.61895
H	-1.80127	4.843062	-3.34594
H	-1.02698	5.390039	-1.8189
O	-1.24214	3.347531	-2.02778

Table S4. Atomistic coordinates of $Li(EC)_4^+$ calculated by using Gaussian 16 with SMD solvation model.

Atom	*x*/(Å)	*y*/(Å)	*z*/(Å)
O	-1.34189	-0.64693	1.146536
O	-2.72386	-1.13302	2.82829
O	1.412777	-2.40906	-2.98852
O	0.821377	-1.33115	-1.12721
O	1.388603	0.811894	1.176015
O	-0.62345	-1.60731	-2.81511
C	-1.75607	-0.43457	2.258993
C	-3.0963	-0.5107	4.066242
H	-3.25322	-1.28779	4.823643
H	-4.03002	0.048308	3.905538
C	2.102173	-0.61404	4.194882
H	2.717827	-1.49373	4.417757
H	1.207305	-0.61373	4.834946
C	0.556388	-1.74795	-2.22783
C	1.781098	0.531091	2.281617
C	2.870091	0.699097	4.245102
H	2.660923	1.298462	5.139287
H	3.956159	0.567147	4.130838
C	-1.9047	0.390733	4.351867
H	-2.18166	1.384941	4.722387
H	-1.16979	-0.06978	5.028961
C	0.734644	-2.90852	-4.14952
H	0.551226	-3.98384	-4.00827
H	1.370736	-2.75293	-5.02919
C	-0.54451	-2.0832	-4.16571
H	-0.48792	-1.21372	-4.83744
H	-1.44386	-2.66867	-4.39146
Li	0.062851	0.034963	0.006651
O	-1.30295	0.525251	3.055674
O	1.679973	-0.67302	2.825924
O	2.37478	1.392337	3.091212
O	-0.65687	1.388414	-1.17026
O	0.551797	1.389245	-3.05637
C	-0.46959	1.768451	-2.2992

C	0.383061	1.913403	-4.38167
H	1.347456	2.290661	-4.742
H	0.032506	1.100277	-5.0344
C	-0.66058	3.001073	-4.17381
H	-1.43398	3.024397	-4.95096
H	-0.22126	4.002517	-4.05546
O	-1.26125	2.620986	-2.92798

Table S5. Atomistic coordinates of $Li(EC)_3^+$@3D-COF−OH calculated by using Gaussian 16 with SMD solvation model.

Atom	*x*/(Å)	*y*/(Å)	*z*/(Å)
C	-9.66463	-2.68757	1.022174
C	-9.36404	-1.92529	-0.11312
H	-9.88524	-2.19675	1.97154
C	-15.99	3.845705	6.458111
C	-11.2584	-0.09266	1.421352
C	-11.9761	0.532241	2.432981
H	-11.7538	-0.8712	0.836073
H	-12.999	0.209528	2.640891
C	-9.76808	-8.97789	-0.0804
O	-10.5114	-8.27883	0.763401
C	-15.3537	3.223483	5.395402
C	-13.9577	3.04245	5.387945
C	-13.3138	2.388923	4.268768
H	-15.9295	2.858935	4.539356
H	-13.9651	2.059643	3.441422
C	-9.86054	-10.3792	-0.09428
C	-9.08583	-11.1241	-0.97361
H	-10.5536	-10.8613	0.599292
N	-9.44564	-6.15936	-0.19491
C	-9.39074	-4.76312	-0.21175
C	-9.6913	-4.07896	0.972736
H	-9.93677	-4.65533	1.868299
H	-10.2816	-7.30788	0.605252
C	-13.9283	4.218127	-7.60158
C	-14.1263	5.59193	-7.55972
H	-14.2724	3.621142	-8.44949
N	-11.9386	2.415477	-4.31804
C	-11.2472	1.768114	-3.29068
C	-11.5659	0.430913	-3.02292
H	-12.3533	-0.05204	-3.60697
H	-12.6184	1.999938	-5.74059
C	-8.09544	-9.11995	-1.8577
C	-8.86867	-8.33688	-0.97985
C	-8.74843	-6.89495	-0.99171

H	-7.40955	-8.61108	-2.54179
H	-8.02523	-6.45998	-1.70192
C	-13.2752	3.563541	-6.54448
O	-13.0941	2.252762	-6.59506
C	-10.9078	-0.27428	-2.01843
C	-9.88903	0.31761	-1.26236
H	-11.2041	-1.30739	-1.82937
C	-8.19658	-10.5024	-1.86298
C	-9.05706	-2.61977	-1.29494
C	-9.07757	-4.00721	-1.35456
H	-8.7952	-2.06365	-2.19835
H	-8.86584	-4.50506	-2.30376
C	14.8029	-4.00586	5.574262
C	14.64269	-2.68229	5.122663
C	13.61529	-2.36155	4.155335
C	15.50322	-1.66419	5.624365
H	14.1338	-4.77517	5.177042
H	12.98906	-3.19539	3.795251
O	15.38878	-0.40755	5.222917
H	-11.6185	2.900974	5.659061
C	-13.8429	4.140378	7.56055
C	-15.222	4.30168	7.539979
H	-13.2428	4.495697	8.401663
N	-12.0363	2.218843	4.239129
C	-11.3849	1.552517	3.197807
C	-10.0584	1.90457	2.919299
H	-9.58727	2.696662	3.506747
C	11.70008	0.688653	1.063813
C	10.45625	0.044141	0.984887
H	11.92526	1.524821	0.398372
C	7.751891	10.60245	0.160488
C	9.588935	2.876052	0.635757
C	9.397545	4.238932	0.427247
H	9.922987	2.545276	1.62109
H	9.613903	4.93801	1.238552
C	-0.42796	-1.27145	1.586377
C	-1.2493	-0.54074	0.711325
C	-2.68935	-0.70019	0.770144

H	-0.89713	-1.95333	2.301918
H	-3.08522	-1.41698	1.508485
N	3.907236	-0.4877	1.275973
C	5.249283	-0.27757	0.911611
C	6.176943	0.113503	1.887954
H	5.837678	0.279365	2.912167
C	7.511126	0.312516	1.555028
C	7.987054	0.089014	0.253847
H	8.196718	0.649664	2.335504
C	16.49092	-2.00922	6.561341
C	16.62258	-3.32369	6.98929
C	15.7806	-4.33385	6.500568
H	17.14644	-1.22138	6.93998
N	13.45485	-1.1572	3.724697
C	12.44355	-0.80601	2.826487
C	12.66835	0.286292	1.975653
C	10.24103	-1.04771	1.837197
C	11.20812	-1.46611	2.74659
H	13.62656	0.809189	2.028242
H	9.294759	-1.5911	1.802217
H	10.98565	-2.30098	3.415344
H	14.63193	-0.39213	4.554009
C	-13.1868	3.510933	6.490154
O	-11.8709	3.364899	6.520316
C	7.904444	9.25641	0.454314
C	8.275611	8.331739	-0.54003
C	8.428275	6.930583	-0.21214
H	7.739749	8.888289	1.471499
H	8.232628	6.637367	0.833216
C	-9.34937	1.274069	1.899836
C	-9.92602	0.250564	1.138462
H	-8.32566	1.59697	1.703101
C	0.952949	-1.14593	1.563536
O	1.737367	-1.85917	2.40537
C	-6.96967	-0.51131	1.12598
C	-5.58273	-0.46192	1.167323
H	-7.50831	-0.7935	2.033781
H	-5.06791	-0.67174	2.107805

C	-6.94684	0.117964	-1.18733
C	-7.68636	-0.20942	-0.04441
H	-7.45546	0.335558	-2.12786
C	-13.6844	6.356074	-6.46928
C	-9.57933	1.660378	-1.53431
C	-10.2321	2.374411	-2.53086
H	-8.80488	2.166873	-0.95301
H	-9.93506	3.406469	-2.7319
C	14.62108	-3.26449	-6.45598
C	13.33114	-3.46766	-5.9305
C	12.83662	-2.62103	-4.86625
C	12.52092	-4.51586	-6.45325
H	15.23006	-2.45586	-6.04063
H	13.5162	-1.83435	-4.4967
O	11.30388	-4.74379	-5.98349
C	8.336122	10.15437	-2.15676
C	7.970957	11.04235	-1.15357
H	8.506409	10.49434	-3.18109
N	8.761441	6.066533	-1.10893
C	8.960543	4.714116	-0.81848
C	8.734839	3.78178	-1.8421
H	8.394512	4.139371	-2.81714
C	9.653391	-1.7444	-1.46057
C	9.970476	-0.38484	-1.3193
H	8.957655	-2.21738	-0.76416
C	-0.6578	0.345314	-0.2289
O	-1.38977	1.054991	-1.0785
C	7.052982	-0.29793	-0.71475
C	5.709318	-0.48182	-0.39712
H	7.369489	-0.46627	-1.74591
H	5.015192	-0.81933	-1.17083
H	8.888669	7.035388	-2.4139
C	-13.0391	5.721175	-5.41955
C	-12.8211	4.330562	-5.43356
C	-12.144	3.688099	-4.32761
H	-12.6847	6.294169	-4.55735
H	-11.8282	4.335357	-3.49187
H	11.1508	-4.06331	-5.25289

C	8.919076	2.422645	-1.61981
C	9.368523	1.938008	-0.38214
H	8.704338	1.726165	-2.43316
C	13.03139	-5.3198	-7.48543
C	14.30709	-5.09276	-7.98496
C	15.1137	-4.0642	-7.47543
H	12.40206	-6.12107	-7.87995
N	11.66191	-2.79452	-4.36454
C	11.1389	-1.9663	-3.36782
C	10.20997	-2.51707	-2.47254
C	10.89906	0.157103	-2.21842
C	11.47102	-0.6104	-3.22895
H	9.942305	-3.57212	-2.57078
H	11.18931	1.206504	-2.13875
H	12.16611	-0.14008	-3.9285
C	8.494826	8.788418	-1.87126
O	8.841309	7.951506	-2.83705
H	-2.34887	0.811661	-0.89023
C	0.73436	0.452518	-0.24967
C	1.554975	-0.26639	0.630397
C	2.993269	-0.06751	0.479595
H	1.192207	1.123824	-0.98023
H	3.266849	0.519271	-0.41412
N	-3.45677	-0.03838	-0.02439
C	-4.85057	-0.12194	0.016632
C	-5.55616	0.175078	-1.15607
H	-4.99689	0.440304	-2.05676
C	-9.21666	-0.39131	-0.07006
C	9.445495	0.42214	-0.11559
H	14.68524	-5.73007	-8.78933
H	16.11562	-3.89734	-7.87684
H	7.46511	11.3114	0.940194
H	7.853375	12.10208	-1.39712
H	17.39817	-3.57065	7.719754
H	15.89524	-5.36381	6.84534
H	-17.0738	3.980273	6.454233
H	-15.7133	4.794036	8.383989
H	-9.17421	-12.2142	-0.96903

H	-7.59321	-11.1003	-2.54946
H	-14.6365	6.082124	-8.39367
H	-13.8476	7.435779	-6.44885
H	1.215874	-2.49944	2.924692
Li	3.559177	-1.52908	3.147306
C	3.419647	1.083165	4.544178
O	2.172194	1.406059	4.23454
C	1.96962	2.805772	4.475377
C	3.139192	3.153134	5.384422
O	4.061839	2.086652	5.121963
O	3.916674	0.006077	4.32829
H	0.988813	2.950184	4.944031
H	2.003262	3.332171	3.510134
H	2.87601	3.129339	6.452289
H	3.618983	4.108606	5.14079
C	5.067929	-4.08861	3.144102
O	6.216427	-4.6876	3.415883
C	5.972801	-6.05534	3.770815
C	4.561747	-6.28645	3.249158
O	4.055778	-4.95045	3.12149
O	4.957275	-2.90792	2.936116
H	6.048033	-6.14827	4.864217
H	6.728078	-6.68938	3.291192
H	3.922859	-6.84611	3.942818
H	4.541829	-6.76105	2.25721
C	2.135723	-3.66672	5.031147
O	2.229254	-4.67562	5.8809
C	1.041564	-5.47846	5.812353
C	0.443818	-5.07613	4.473425
O	1.042398	-3.78724	4.260638
O	2.905593	-2.75202	4.945641
H	0.396924	-5.21822	6.664872
H	1.322574	-6.53675	5.867652
H	-0.64678	-4.9659	4.491907
H	0.744333	-5.73933	3.64901

Table S6. Atomistic coordinates of EC calculated by using Gaussian 16 with SMD solvation model.

Atom	*x*/(Å)	*y*/(Å)	*z*/(Å)
O	1.121474	-2.83711	-2.41317
O	0.016472	-0.88627	-2.31659
O	-1.07316	-2.84581	-2.22176
C	0.02117	-2.08047	-2.31713
C	0.779411	-4.20596	-2.19303
H	1.038059	-4.47494	-1.15674
H	1.344524	-4.83866	-2.88877
C	-0.72036	-4.21172	-2.44321
H	-0.97693	-4.48175	-3.47975
H	-1.28045	-4.8495	-1.74804

References

(1) Xu, Y.; Sun, T.; Zeng, T.; Zhang, X.; Yao, X.; Liu, S.; Shi, Z.; Wen, W.; Zhao, Y.; Jiang, S.; et al. Symmetry-breaking dynamics in a tautomeric 3D covalent organic framework. *Nat. Commun.* **2023**, *14* (1), 4215.

(2) Huang, S. Y.; Kavan, L.; Exnar, I.; Grätzel, M. Rocking Chair Lithium Battery Based on Nanocrystalline TiO2 (Anatase). *J. Electrochem. Soc.* **2019**, *142* (9), L142-L144.

(3) Watanabe, H.; Omoto, S.; Hoshi, Y.; Shitanda, I.; Itagaki, M. Electrochemical impedance analysis on positive electrode in lithium-ion battery with galvanostatic control. *J. Power Sources* **2021**, *507*, 230258.

(4) Chen, K.-H.; Wood, K. N.; Kazyak, E.; LePage, W. S.; Davis, A. L.; Sanchez, A. J.; Dasgupta, N. P. Dead lithium: mass transport effects on voltage, capacity, and failure of lithium metal anodes. *J. Mater. Chem. A* **2017**, *5* (23), 11671-11681.

(5) Koo, D.; Kwon, B.; Lee, J.; Lee, K. T. Asymmetric behaviour of Li/Li symmetric cells for Li metal batteries. *Chem. Commun.* **2019**, *55* (65), 9637-9640.

(6) Sasaki, Y.; Hosoya, M.; Handa, M. Lithium cycling efficiency of ternary solvent electrolytes with ethylene carbonate—dimethyl carbonate mixture. *J. Power Sources* **1997**, *68* (2), 492-496.

(7) Rong, H.; Xu, M.; Xie, B.; Huang, W.; Liao, X.; Xing, L.; Li, W. Performance improvement of graphite/LiNi0.4Co0.2Mn0.4O2 battery at high voltage with added Tris (trimethylsilyl) phosphate. *J. Power Sources* **2015**, *274*, 1155-1161.

(8) Johnson, C. S. Charging Up Lithium-Ion Battery Cathodes. *Joule* **2018**, *2* (3), 373-375.

(9) Wang, L.; Huang, B.; Xiong, W.; Tong, M. e.; Li, H.; Xiao, S.; Chen, Q.; Li, Y.; Yang, J. Improved solid-state synthesis and electrochemical properties of LiNi0.6Mn0.2Co0.2O2 cathode materials for lithium-ion batteries. *J. Alloys Compd.* **2020**, *844*, 156034.

(10) Gaberšček, M. Understanding Li-based battery materials via electrochemical impedance spectroscopy. *Nat. Commun.* **2021**, *12* (1), 6513.

(11) Hallemans, N.; Widanage, W. D.; Zhu, X.; Moharana, S.; Rashid, M.; Hubin, A.; Lataire, J. Operando electrochemical impedance spectroscopy and its application to commercial Li-ion batteries. *J. Power Sources* **2022**, *547*.

(12) Hohenberg, P.; Kohn, W. Inhomogeneous Electron Gas. *Phys. Rev. B* **1964**, *136* (3b), B864-B871.

(13) Kohn, W.; Sham, L. J. Self-Consistent Equations Including Exchange and Correlation Effects. *Phys. Rev.* **1965**, *140* (4a), 1133-1138.

(14) Kresse, G.; Furthmüller, J. Efficiency of ab-initio total energy calculations for metals and semiconductors using a plane-wave basis set. *Comp Mater Sci* **1996**, *6* (1), 15-50.

(15) Kresse, G.; Furthmuller, J. Efficient iterative schemes for ab initio total-energy calculations using a plane-wave basis set. *Phys Rev B Condens Matter* **1996**, *54* (16), 11169-11186.

(16) Perdew, J. P.; Burke, K.; Ernzerhof, M. Generalized Gradient Approximation Made Simple. *Phys. Rev. Lett.* **1996**, *77* (18), 3865-3868.

(17) Kresse, G.; Joubert, D. From ultrasoft pseudopotentials to the projector augmented-wave method. *Physical Review B* **1999**, *59* (3), 1758-1775.

(18) Grimme, S. Accurate description of van der Waals complexes by density functional theory including empirical corrections. *J. Comput. Chem.* **2004**, *25* (12), 1463-1473.

(19) Grimme, S. Semiempirical GGA-type density functional constructed with a long-range dispersion correction. *J Comput Chem* **2006**, *27* (15), 1787-1799.

(20) Grimme, S.; Ehrlich, S.; Goerigk, L. Effect of the damping function in dispersion corrected density functional theory. *J. Comput. Chem.* **2011**, *32* (7), 1456-1465.

(21) Marenich, A. V.; Cramer, C. J.; Truhlar, D. G. Universal solvation model based on solute electron density and on a continuum model of the solvent defined by the bulk dielectric constant and atomic surface tensions. *J Phys Chem B* **2009**, *113* (18), 6378-6396.

(22) Frisch, M. J.; Trucks, G. W.; Schlegel, H. B.; Scuseria, G. E.; Robb, M. A.; Cheeseman, J. R.; Scalmani, G.; Barone, V.; Petersson, G. A.; Nakatsuji, H.; et al. Gaussian 16, Rev. *C* **Gaussian, Inc., 2016**.

(23) Adamo, C.; Barone, V. Toward reliable density functional methods without adjustable parameters: The PBE0 model. *J. Chem. Phys.* **1999**, *110* (13), 6158-6170.

(24) Ernzerhof, M.; Scuseria, G. E. Assessment of the Perdew–Burke–Ernzerhof exchange-correlation functional. *J. Chem. Phys.* **1999**, *110* (11), 5029-5036.

(25) Weigend, F.; Ahlrichs, R. Balanced basis sets of split valence, triple zeta valence and quadruple zeta valence quality for H to Rn: Design and assessment of accuracy. *Phys Chem Chem Phys* **2005**, *7* (18), 3297-3305.

(26) Weigend, F. Accurate Coulomb-fitting basis sets for H to Rn. *Phys Chem Chem Phys* **2006**, *8* (9), 1057-1065.

(27) Borodin, O.; Behl, W.; Jow, T. R. Oxidative Stability and Initial Decomposition Reactions of Carbonate, Sulfone, and Alkyl Phosphate-Based Electrolytes. *J. Phys. Chem. C* **2013**, *117* (17), 8661-8682.

(28) Lu, T.; Chen, F. Multiwfn: a multifunctional wavefunction analyzer. *J Comput Chem* **2012**, *33* (5), 580-592.

(29) BIOVIA, D. S., Discovery Studio Visualizer, 2024, San Diego: Dassault Systèmes, 2024.

(30) Momma, K.; Izumi, F. VESTA 3for three-dimensional visualization of crystal, volumetric and morphology data. *Journal of Applied Crystallography* **2011**, *44* (6), 1272-1276.
